# Photosynthetic Co-production of Succinate and Ethylene in a Fast-Growing Cyanobacterium, *Synechococcus elongatus* PCC 11801

**DOI:** 10.3390/metabo10060250

**Published:** 2020-06-16

**Authors:** Annesha Sengupta, Prem Pritam, Damini Jaiswal, Anindita Bandyopadhyay, Himadri B. Pakrasi, Pramod P. Wangikar

**Affiliations:** 1 Department of Chemical Engineering, Indian Institute of Technology Bombay, Powai, Mumbai 400076, India; annesha.sengupta@iitb.ac.in (A.S.); prempritam14@iitb.ac.in (P.P.); damini.jaiswal@iitb.ac.in (D.J.); 2 Department of Biology, Washington University, St. Louis, MO 63105, USA; anindita@wustl.edu; 3 Department of Energy, Environmental and Chemical Engineering, Washington University, St. Louis, MO 63105, USA; 4 DBT-Pan IIT Center for Bioenergy, Indian Institute of Technology Bombay, Powai, Mumbai 400076, India; 5 Wadhwani Research Center for Bioengineering, Indian Institute of Technology Bombay, Powai, Mumbai 400076, India

**Keywords:** cyanobacteria, metabolomics, CRISPR-Cpf1, ^13^C isotopic labelling, ethylene, succinate

## Abstract

Cyanobacteria are emerging as hosts for photoautotrophic production of chemicals. Recent studies have attempted to stretch the limits of photosynthetic production, typically focusing on one product at a time, possibly to minimise the additional burden of product separation. Here, we explore the simultaneous production of two products that can be easily separated: ethylene, a gaseous product, and succinate, an organic acid that accumulates in the culture medium. This was achieved by expressing a single copy of the ethylene forming enzyme (*efe*) under the control of P_cpcB_, the inducer-free super-strong promoter of phycocyanin β subunit. We chose the recently reported, fast-growing and robust cyanobacterium, *Synechococcus elongatus* PCC 11801, as the host strain. A stable recombinant strain was constructed using CRISPR-Cpf1 in a first report of markerless genome editing of this cyanobacterium. Under photoautotrophic conditions, the recombinant strain shows specific productivities of 338.26 and 1044.18 μmole/g dry cell weight/h for ethylene and succinate, respectively. These results compare favourably with the reported productivities for individual products in cyanobacteria that are highly engineered. Metabolome profiling and ^13^C labelling studies indicate carbon flux redistribution and suggest avenues for further improvement. Our results show that *S. elongatus* PCC 11801 is a promising candidate for metabolic engineering.

## 1. Introduction

Cyanobacteria are being explored as cell factories due to their ability to convert sunlight and atmospheric CO_2_ into a variety of chemicals [1]. Cyanobacteria grow faster than other photosynthetic organisms, are tolerant to stresses and can be engineered genetically to express heterologous pathways. However, processes employing these photoautotrophic prokaryotes may not become commercially viable until the productivities are significantly improved. It has been proposed that the use of fast-growing and robust cyanobacteria as chassis may result in improved carbon capture efficiency and greater product formation rates [2,3,4]. Thus, the quest for the identification of fast-growing cyanobacteria has led to the isolation of three *Synechococcus elongatus* strains, UTEX 2973 [5], PCC 11801 [6] and PCC 11802 [7], and the euryhaline *Synechococcus* sp. PCC 11901 [8] which have significantly higher growth rates than model cyanobacteria such as *Synechocystis* sp. PCC 6803 and *S. elongatus* PCC 7942. In parallel, other significant work includes improvement of synthetic biology toolbox for cyanobacteria by creating libraries of genetic parts, assembly tools, genome editing tools and transcription modification tools [9]. While much of this work has been with model cyanobacteria, characterisation and optimisation of the parts and tools in the newly isolated fast-growing strains is gaining momentum [3,4,10,11]. To exemplify, the improved markerless genome editing tool, CRISPR-Cpf1, and gene assembly methods, CyanoGate, have been optimised for *S. elongatus* UTEX 2973 [9,12,13]. Besides the synthetic biology toolbox, improved omics techniques and metabolic models are being developed to aid the identification of bottlenecks in cyanobacteria [2,14,15,16]. These tools are expected to speed up metabolic engineering of cyanobacteria that will have improved productivity.

Cyanobacteria have been engineered to produce a number of products including succinate and ethylene [17]. While the former is an important platform chemical with applications in the pharmaceutical and agricultural industry, the latter is a gaseous product that serves as a petroleum feed-stock [17]. Succinic acid is a natural intermediate of the tricarboxylic acid (TCA) cycle. However, in cyanobacteria, the TCA cycle was thought to be incomplete due to the absence of the α-ketoglutarate dehydrogenase enzyme. That understanding has changed now with the discovery of two genes 2-oxoglutarate decarboxylase (*OgdA*) and succinate semialdehyde dehydrogenase (*SsaD*) in *Synechococcus* sp. PCC 7002 which complete the TCA cycle [18]. Indeed, heterologous expression of these two genes has been shown to result in enhanced production of succinate in *S. elongatus* PCC 7942, albeit at the cost of growth [19]. The growth retardation was thought to be a result of depletion of TCA cycle intermediates due to secretion of succinate. The phenotype was rescued, concomitantly achieving a succinate productivity of 10.7 mg·(g dry cell weight)^−1^·h^−1^ with the overexpression of phosphoenolpyruvate carboxylase (*ppc*) and citrate synthase (*gltA*) [19]. Merely overexpressing the *ppc* and *gltA* genes without the *OgdA* and *SsaD* genes did not result in appreciable succinate titres [20,21]. A temperature-dependent dark anoxic succinate production was reported in *Synechocystis* sp. PCC 6803 with overexpression of *ppc* and knockout of acetate kinase gene. While this study achieved high volumetric titres, the specific productivity was very low [22]. Thus, striking a balance between the volumetric and specific productivity would help improve the carbon partitioning toward the product of interest [23]. Unlike succinate, ethylene is not naturally produced in cyanobacteria. Cyanobacteria have been engineered to produce ethylene from 2-oxoglutarate by expressing a single enzyme, the ethylene forming enzyme (EFE) from *Pseudomonas syringae* [24,25,26,27,28]. The highest reported ethylene productivity of 2463 µL/L/h/OD_730nm_ was observed with four copies of *efe* gene which partially deleted the global transcription factor, nitrogen control A (NtcA) [26]. The production was initially speculated to be limited by EFE [25,26]. However, recently, the rate-limiting step was identified to be the anaplerotic reaction and overexpression of *ppc* enhanced carbon assimilation increasing the productivity [29]. Table 1 summarises the various metabolic engineering strategies adopted for photoautotrophic production of succinate and ethylene and the specific product formation rates. The rate values reported in different units were converted to common units (Table S2).

Heterologous expression of metabolic pathways is expected to re-distribute the carbon flux. Metabolic control analysis (MCA) has been used to show that the flux alteration is dependent on the flux control coefficient of the enzyme(s). Furthermore, MCA can be used to identify the flux controlling enzymes so that the metabolic engineering efforts can be focused on those enzymes [34,35]. Likewise, metabolomics and metabolic flux analysis can be employed to identify the potential bottlenecks and rationally engineer the strain to enhance productivity, as has been demonstrated for butyraldehyde production in *S. elongatus* PCC 7942 [36,37]. Similarly, Xiong et al. performed isotopic non-stationary ^13^C metabolic flux analysis (INST-MFA) on an ethylene producing *Synechocystis* sp. PCC 6803 strain to obtain a better insight into the cyanobacterial metabolism. A greater carbon flux was reported to be directed towards the heterologous ethylene pathway leading to a larger than usual flux through the TCA cycle [30].

While the literature reports on heterologous production in cyanobacteria have primarily focused on single product formation, co-production of compounds might be more economical, provided the product separation cost can be minimised. The well-known EFE is a multi-product enzyme catalysing the formation of four products, ethylene, succinate, guanidine and (S)-1-Pyrroline-5-carboxylate (P5C). While ethylene is gaseous, the remaining three products remain in the liquid phase [38,39]. P5C is utilised for arginine biosynthesis, which acts as a substrate for the reaction [40]. Guanidine, on the other hand, was co-produced with ethylene showing a titre of 586.5 mg/L after seven days of cultivation [28]. However, no extracellular succinate accumulation was observed in the *efe* expressing *Synechocystis* sp. PCC 6803 strain [30]. Since *S. elongatus* PCC 11801 has been reported to exhibit high tolerance towards abiotic factors [6], we explored the potential of this strain to simultaneously produce succinate and ethylene.

*S. elongatus* PCC 11801 was engineered to co-produce ethylene and succinate by expressing the codon-optimised *efe* gene under the control of a strong truncated cpcB promoter of *S. elongatus* PCC 7942 (P_cpcB7942_) [41]. The CRISPR-Cpf1 based genome-editing strategy was employed for the knock-in of the promoter-gene-terminator cassette at the neutral site (NS) I of *S. elongatus* PCC 11801. Thus, this is the first report on the use of CRISPR-based genome editing of *S. elongatus* PCC 11801. The growth conditions for the production of succinate and ethylene were optimised. A detailed metabolome profiling and ^13^C labelling studies were performed to obtain clues about the potential metabolic control points.

## 2. Results and Discussion

The co-production of succinate and ethylene by expressing *efe* gene has been reported to be thermodynamically feasible [42]. Further, both the products do not show toxic effect on cyanobacteria [19,25]. It is well known that the cyanobacterial TCA cycle is more active under low light or darkness than under high light [43,44]. Since the EFE enzyme utilises the TCA cycle intermediate, 2-oxoglutarate, we employed a promoter that would be highly active under low light. The characterisation of cpcB promoter from *S. elongatus* PCC 7942 (P_cpcB7942_) showed that it is one of the super-strong promoters exhibiting higher activity under low light and elevated CO_2_ conditions [41]. The promoters have been observed to be highly portable among phylogenetic close neighbours [6,41]. A CRISPR-based, markerless genome editing approach was used to develop the *efe*-expressing strain of *S. elongatus* PCC 11801. Antibiotic marker-free recombinant strains are likely to be safer for use in large-scale, outdoor cultivations as they do not pose the risk of spread of antibiotic resistance [45]. Further, the CRISPR-based techniques speed up the chromosomal segregation in cyanobacteria that contain multiple copies of chromosome [3]. Each copy must carry the genetic modification of interest in order to obtain a stable recombinant strain. Moreover, additional genetic modifications can be performed once a stable, marker-free strain is obtained. We chose the CRISPR-Cpf1 technique over CRISPR-cas9 due to the various advantages that the former offers in cyanobacteria [12]. Chiefly, Cpf1, a type V-A nuclease, is significantly less toxic to cyanobacteria than cas9. Cpf1 carries out a 5-bp staggered double stranded break 17 nucleotides downstream from the PAM sequence, which can then be repaired with a repair template that comprises of the homology arms and the desired payload [12].

We used the Cpf1-harbouring, self-replicating plasmid pSL2680 that alleviates the need to integrate the nuclease into the chromosome, to construct the editing plasmid pSL3338 by incorporating the guide RNA and the repair template. The editing plasmid was cured after complete chromosomal segregation to obtain a truly markerless recombinant strain that is devoid of the Cpf1 nuclease and antibiotic resistance genes. This strategy allowed us to create the *efe* expressing strain, *S. elongatus* PCC 11801:NSI::P*_cpcB_*-*efe*-T*_rrnB_* strain (henceforth SL3338) (Figure S1). NSI, locus tag DOP62_03525, a gene with ~84% identity with NSI of *S. elongatus* PCC 7942, was chosen as the site for chromosomal integration in *S. elongatus* PCC 11801 based on previous reports [6,7]. Complete segregation of SL3338 strain was ascertained by gel electrophoresis (Figure S2a). As a first step, the expression of *efe* gene was confirmed by isolating the RNA sample and by performing reverse transcriptase PCR (RT-PCR) and gel electrophoresis. Prominent bands corresponding to the *efe* gene, which was absent in the wild type strain and the negative control that lacked the reverse transcriptase step, confirmed the expression (Figure S2b). Next, it was of interest to optimise the conditions for the co-production of ethylene and succinate. Note that there is significant difference in the methods to be used for the determination of the two products. Succinate accumulates in the culture medium and can be assayed either with an enzymatic reaction-based, spectrophotometric detection kit or via GC-MS upon appropriate derivatisation. For the determination of ethylene production rate, the culture first needs to be transferred to an airtight bottle followed by sampling of the headspace at regular intervals and assay via GC-FID. Thus, the growth conditions were first optimised for the production of succinate and then ethylene production rates were estimated for some of the conditions. This strategy was adopted primarily due to the relative ease of succinate detection.

### 2.1. Optimising Growth Condition for Improved Succinate Production in the Recombinant Strain

**Figure 1:**
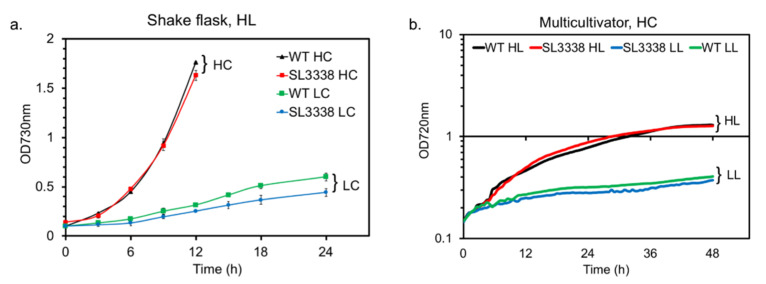
The growth profile of the recombinant and wild type strains: (**a**) The comparison of growth profile of strains cultivated with either ambient CO_2_ (LC) or 1% CO_2_ (HC) under high light intensity (300 µE·m^−2^·s^−1^) in shake flask (SF), agitation of 120 rpm. (**b**) The growth profile of strains in Multi-Cultivator (MC 1000-OD) with 1% CO_2_ bubbled at a rate of 100 sccm under HL (300 µE·m^−2^·s^−1^) or LL (20 µE·m^−2^·s^−1^) intensities.

We characterised the growth of the recombinant *S. elongatus* PCC 11801 (SL3338) strain under low and high light (LL and HL), ambient and 1% CO_2_ (LC and HC) and in shake flask (SF) or multi-cultivator (MC). In SF, while SL3338 showed growth defect with ~20% reduction in biomass accumulation under LC-HL (Figure 1a), the extent of growth retardation was negligible under HC-HL (Figure 1a). On the contrary, under HC-LL, SL3338 showed lower growth for the initial 24 h or so but eventually accumulated biomass that was comparable to that of HL-HC (Figure S3). Under both high and low light, the WT and SL3338 strains accumulated ~6–7 g DCW in 72–120 h under HC. Thus, similar to earlier reports, the expression of *efe* did not impose a severe metabolic burden under HC and showed no significant growth retardation. Moreover, the photosynthetic efficiency, as a function of the Fv/Fm was observed to be not compromised between the strains (Table S3). Unlike SF, growth in MC 1000-OD under 20 µE·m^−2^·s^−1^ with bubbled 1% CO_2_ showed significantly retarded growth for both strains (Figure 1b). However, high light intensity ensured higher growth and biomass accumulation in MC 1000-OD with minimum growth defect between wild type and recombinant strains (Figure 1b). The best growth conditions might not be the best production conditions as product formation is also dependent on the transcriptional and translational rates of protein production.

**Figure 2:**
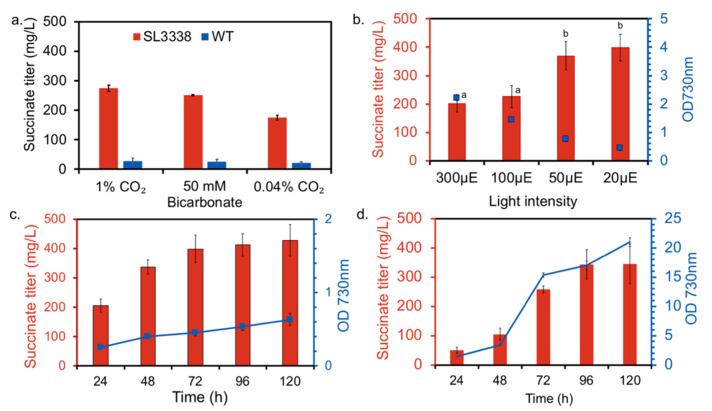
Effect of growth conditions on succinate titres: (**a**) Effect of bicarbonate addition: Succinate levels measured at 120 h for strains SL3338 and WT with or without the addition of 50 mM bicarbonate. Flasks were incubated under ambient air, light intensity of 200 µE·m^−2^·s^−1^ and agitation of 120 rpm. (**b**) Effect of light intensity on growth and succinate titres in multicultivator: the SL3338 strain was grown under continuous illumination at the specified light intensity and OD_730_ and succinate titres measured at 120 h. The SL3338 strain was grown in MC 1000-OD with 1% CO_2_ bubbled at 100 sccm per tube. (**c**) Time course measurement of OD_730nm_ and succinate titres for the SL3338 strain grown in multicultivator under 20 µE·m^−2^·s^−1^ light and 1% CO_2_ bubbled at a rate of 100 sccm/tube (the LL-HC condition). (**d**) Time course measurement of OD_730nm_ and succinate titres for the SL3338 strain grown in a shake flask under 20 µE·m^−2^·s^−1^ light and 1% CO_2_ (LL-HC) in shaker incubator with an agitation of 120 rpm. All growth experiments were carried out in BG11 medium at 38 °C. Succinate in the extracellular medium measured with the Succinate estimation kit from Megazyme. Error bars correspond to standard error of mean (SEM) of the biological replicates (n = 3).

Succinate was estimated to optimise the growth conditions as succinate is excreted into the culture medium and easily detected. While no experimental reports are available on extracellular succinate accumulation by *efe*-expressing cyanobacteria, the engineered strain of this study (SL3338) showed ~7-fold higher extracellular succinate level in shake flask as compared to wild type after 120 h of SF cultivation under LC-HL. The succinate productivity may have been limited by the availability of the carbon substrate as daily addition of 50 mM sodium bicarbonate to the culture or growth under 1% CO_2_ have improved the succinate productivity by 40% (Figure 2a). Likewise, an improved productivity was observed under HC compared to LC (data not shown). Thus, all the subsequent trials for succinate productivity were carried out under 1% CO_2_ (or HC), both in SF and MC1000-OD. Next, it was of interest to identify the optimal light intensity for product formation. In going from the light intensity of 300 to 20 µE·m^−2^·s^−1^, the biomass levels decreased but the product titre increased yielding the highest specific productivity at 20 µE·m^−2^·s^−1^ (Figure 2b). Therefore, the increased productivity under LL-HC may be a combined effect of the high promoter activity of P_cpcB7942_ [41] and a greater TCA cycle activity in at low light [43,44]. Note that α-ketoglutarate, a key precursor of the EFE enzyme, is an intermediate of the TCA cycle and its availability is expected to affect the product formation.

The availability of oxygen is essential for EFE catalysed reaction, therefore different cultivation flasks were tested to investigate the effect of oxygen under the production condition. Although the biomass accumulation in the bottle was lower than in shake flasks, no significant improvement in succinate titre was observed in baffled flasks as compared to unbaffled flasks or bottles (Figure S4). Cyanobacterial cultures exhibit super-saturated levels of dissolved oxygen under photoautotrophic conditions [46,47] and this may explain the lack of effect of baffles on succinate production.

Fumarate is a product of both the oxidative and reductive arm of the TCA cycle. Since excess succinate can potentially get converted into fumarate via the oxidative arm [30], fumarate can potentially accumulate as an undesirable by-product. We observed accumulation of 30–40 mg/L fumarate in SL3338 compared to 5–10 mg/L for the WT cultures under identical conditions (data not shown). We hypothesise that the levels of fumarate can be minimised by knocking out the enzyme succinate dehydrogenase [21]. However, for ethylene production, the use of fumarate as an endogenous substrate has been predicted to be thermodynamically advantageous to enhance the driving force towards ethylene [42]. Therefore, we propose the Sdh knockdown as the future work for improved succinate yield and reduced fumarate side product.

### 2.2. Nitrate Limitation Positively Affected Succinate Production

**Figure 3:**
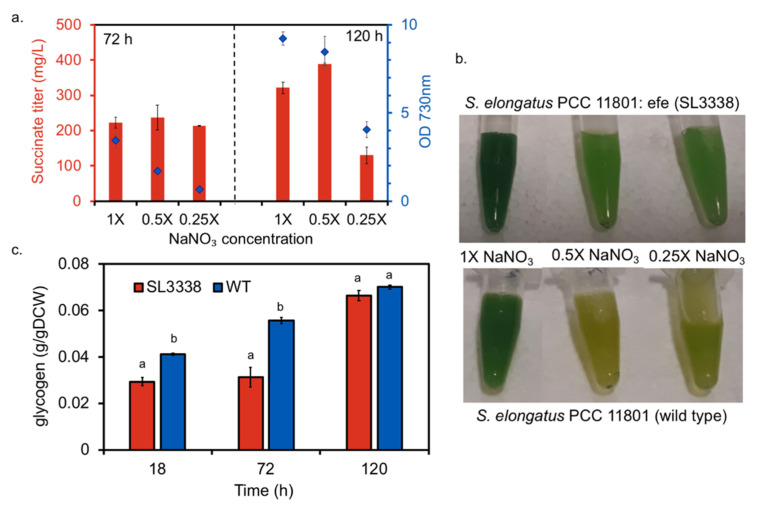
The effect of nitrate limitation on succinate production: (**a**) The OD_730nm_ and succinate titres were measured for the SL3338 strain at 72 and 120 h under the LL-HC in 25 mL shake flasks with 7 mL BG-11 media and varying NaNO_3_ concentrations. The 1× nitrate is equivalent to 1.5 g/L. The error bars correspond to SEM from the biological replicates (n = 3). (**b**) The non-bleaching phenotype exhibited by the recombinant strain as compared to wild type under nitrate limitation. Refer to Section 3.5 for experimental details. (**c**) Glycogen content measured as per the protocol reported in [6]. For glycogen estimation, the cultures were grown at 38 °C under low light intensity (20 µE·m^−2^·s^−1^) in shaker incubator aerated with 1% CO_2_ to different cell densities (OD_730nm_). The error bars for the glycogen data correspond to SEM from three biological replicates (and two technical replicates for each biological replicate) (n = 3) and the letters a and b denote statistically different values for each category (*p* < 0.05) using t-test.

Nitrogen deprivation is known to induce overflow metabolism in cyanobacteria apart from causing an increase in the intracellular 2-oxoglutarate pool [48,49]. It was of interest to test the effect of nitrate on succinate productivity as 2-oxoglutarate is a key precursor for the EFE enzyme. Previous reports have shown that growth in either BG11_0_ or 1 mM NaNO_3_ supplemented BG11 was highly retarded with insignificant ethylene production [25,33]. Therefore, the effect of nitrate on product formation was investigated in the recombinant and wild type strains under different nitrate (NaNO_3_) concentrations, viz., 0.25×, 0.5× and 1× (17 mM) NaNO_3_. Though there was no change in the succinate accumulation in recombinant strain across the conditions after 72 h, the succinate level in 0.5× nitrate concentration was higher than in 1× nitrate after 120 h (Figure 3a). Further reduction in nitrate concentration led to reduced succinate titres (Figure 3a). While growth was retarded under nitrate limited state, the recombinant strain did not show bleached phenotype unlike the wild type strain (Figure 3b). Generally, glycogen deficient strains exhibit a non-bleaching phenotype during nitrogen starvation as a result of an unknown mechanism [48,50]. Recently, a correlation between glycogen deficiency and alternation in the adenylate energy charge has been shown which in turn leads to higher accumulation of organic acids under nitrogen deplete condition [51]. The energy charge level in the cell has been reported to be associated with cyanobacterial growth and partitioning of carbon. We hypothesised that non-bleaching phenotypes is triggered as a result of difference in energy charge distribution between wild type and mutant cells [52]. Since the recombinant strain showed lower glycogen accumulation than wild type under both high and low light intensities (Figure 3c and Figure S5), similar metabolic re-arrangement might have triggered non-bleaching phenotype in the SL3338 strain.

### 2.3. Efficient Carbon Partitioning Between Product Formation and Biomass

The recombinant and wild type strains were cultivated to measure the levels of succinate under more controlled conditions in MC 1000-OD with bubbling 1% CO_2_. While cultivation under high light intensity of 300 µE·m^−2^·s.^−1^ exhibited higher growth, the production was restricted to 250 mg/L (200 mg/gDCW) of succinate in 120 h (Figure S6). On the contrary, the combination of low light and 1% CO_2_ (LL-HC) highly impaired growth of both wild type and recombinant, but showed significantly higher succinate titres (Figure 2c). The peak specific production rate of 121.1 mg/gDCW/h was observed, which is much higher than the reported rates (Table 1).

**Figure 4:**
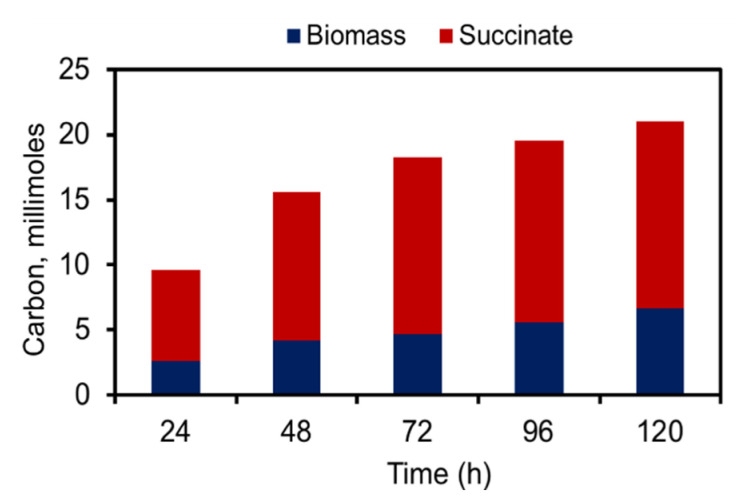
Carbon partitioning: The carbon distribution between product of interest and biomass accumulated for both wild type and recombinant strains when cultivated under LL-HC in multicultivator with 1% CO_2_-mixed air bubbled.

Optimal carbon partitioning would ensure a perfect balance between biomass accumulation and product formation [23]. Several of the metabolic engineering studies in cyanobacteria suffer from low specific productivity as the recombinant strain exhibits a substantial increase in biomass [19,22,25]. There have been efforts to balance the carbon flow between biomass and product accumulation by downregulating the citrate synthase gene, which arrests growth and diverts greater amounts of carbon towards product formation [23]. This strategy of targeting citrate synthase might not be applicable for our study as we focus on increasing flux through TCA cycle. Besides genetic modifications, external factors such light, temperature and pH have also been observed to modulate total intracellular carbon flux in organism [53]. In this study, a similar carbon partitioning was facilitated in the recombinant strain when cultivated in MC 1000-OD under 20 µE·m^−2^·s^−1^ light intensity and 1% CO_2_. The combination of low light and high CO_2_ arrested growth, ensuring carbon flow towards the product of interest, thereby enhancing the specific productivity and carbon partitioning toward the product (Figure 2c and Figure 4). The carbon partitioning was calculated using the equations described below (Equations (1) and (2)):

(1) $$Succinate\;\left(C-moles\right)=\frac{concentration\;of\;succinate\;\left(\frac{g}{L}\right)}{Molecular\;wt\;\left(\frac{g}{mole}\right)\;}*4$$

(2) $$Biomass\;\left(C-moles\right)=\frac{Dry\;cell\;wt\;\left(\frac{g}{L}\right)}{Molecular\;wt\;\left(\frac{g}{mole}\right)\;}$$

where the molecular wt. of succinate is 118.09 g/M while that of biomass (with chemical composition of CH_1.8_O_0.5_N_0.15_) is 23.9 g/M. 

Unlike MC, the shake flask showed much greater carbon diverted towards biomass formation under conditions of LL-HC maintained in a shaker incubator (Figure 2d). MC represents a more carbon saturated state because 1% CO_2_ is bubbled into the culture than shake flask where mixing is facilitated by surface aeration [41]. Since bicarbonate has been reported to be used as a buffering agent, 1% CO_2_ bubbling in MC might provide an improved buffering system than 1% CO_2_ in chamber. Moreover *S. elongatus* PCC 11801 exhibits better growth rate under unbuffered media (Figure S7) in shake flask than in MC under 1% CO_2_ [6]. Apart from the differences in the pH, the difference in the light penetration in the cultures due to the different arrangement of the LED lights in a shaker versus the MC might also affect the growth pattern. Thus, the reduction in biomass accumulation observed in MC 1000-OD might be a combinatorial effect of changed external growth conditions.

### 2.4. Co-Production of Ethylene in S. elongatus PCC 11801-efe Strain

**Figure 5:**
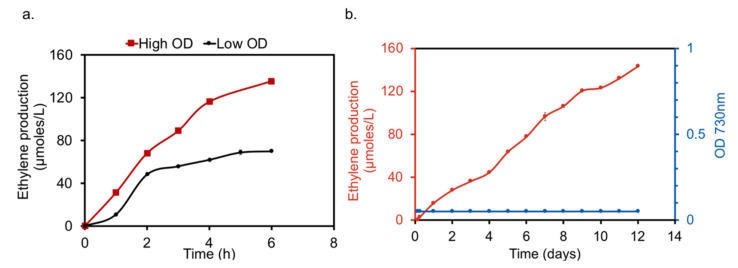
Ethylene production under low light intensity: (**a**) Estimation of ethylene accumulation over a period of 6 h in closed air tight bottles of 120 mL under LL, when the recombinant strain was inoculated with either an OD_730nm_ of 0.2 or 2.15. (**b**) Steady accumulation of ethylene using very low cell OD_730nm_ of 0.05 with the addition of 50 mM sodium bicarbonate at t = 0. Ethylene was measured every day for a period of 12 days. The ethylene accumulation was performed in closed air tight bottles of 120 mL under LL. The head space for ethylene accumulation was maintained at 1:5 (20 mL culture and 100 mL head space). Error bars correspond to SEM of the biological replicates (n = 3).

Ethylene is the primary product of the EFE mediated reaction and stoichiometrically two moles of ethylene are produced from three moles of 2-oxoglutarate [31]. Since it is a gaseous product, airtight bottles were used for ethylene production. The recombinant and the wild type strains were grown in a shake flask or MC 1000-OD under LL-HC, the conditions that were optimised for succinate production and were inoculated with different biomass in bottles under ~20 µE·m^−2^·s^−1^ light intensity. Ethylene accumulated in the headspace was estimated every hour using GC-FID. While the wild type strain showed no ethylene accumulation, the recombinant strain showed ethylene production which increased when incubated with higher biomass (Figure 5a). Although the accumulation was observed to be saturated within 8 h (Figure 5a), the same biomass could be utilised multiple times for ethylene production without compromising the potential of the strain (Figure S8). The early saturation in ethylene accumulation was overcome with very low inoculum (OD_730nm_ 0.05) culture incubated in a bottle under low light and 50 mM sodium bicarbonate. The low inoculum ensured better light penetration as compared to higher cell density cultures and further supplementation with bicarbonate might have drastically changed the growth condition thereby facilitating steady increase in ethylene accumulation. Although a steady increase in ethylene accumulation was observed over a period of 12 days with negligible increment in biomass, the rate of increase was slower in comparison to higher biomass (Figure 5b). As observed with respect to succinate, production in the bottle showed steady maintenance of biomass and diverting more carbon towards the product of interest, facilitating better partitioning of carbon than shake flasks (Figure S9). Therefore, under optimal production conditions, the SL3338 strain was able to co-produce 338.26 µmole/gDCW/h of ethylene using a single copy of *efe* gene, which is by far significant specific productivity of ethylene with minimal modifications (Table 1). A detailed comparison of the product levels along with the reported conditions is provided in Table S2. 

### 2.5. Metabolic Profiling and ^13^C Labelling

Metabolomics and ^13^C isotopic carbon labelling can provide some key insights into a strain engineering workflow. Metabolite pool sizes provide a static snapshot of the metabolism, which in turn may be dependent on the net rate of synthesis of each metabolism. Mapping the changes in metabolite levels can potentially provide unique insights in the differential metabolic activity between the wild type and the engineered strain. ^13^C isotope labelling studies, on the other hand, can reveal additional information on the changes in the reaction rates in metabolic pathways [22]. To that end, we utilised orthogonal metabolomics techniques, viz., measurement of relative pool sizes and the dynamic ^13^C isotopic enrichment of the various intermediate metabolites of the central carbon metabolism, amino acids, sugar nucleotides and cofactors. Since low light intensity ensured the highest productivity of succinate, the metabolomics experiments were performed under similar conditions in shake flask for recombinant and wild type strains. The results of metabolite profiling and ^13^C enrichment were analysed together to identify possible redistribution of the carbon. For example, a slower ^13^C enrichment can result either from a slower rate of reaction or from a larger pool size. However, a slower reaction rate can be unequivocally concluded from a smaller pool size together with slower labelling rate.

**Figure 6:**
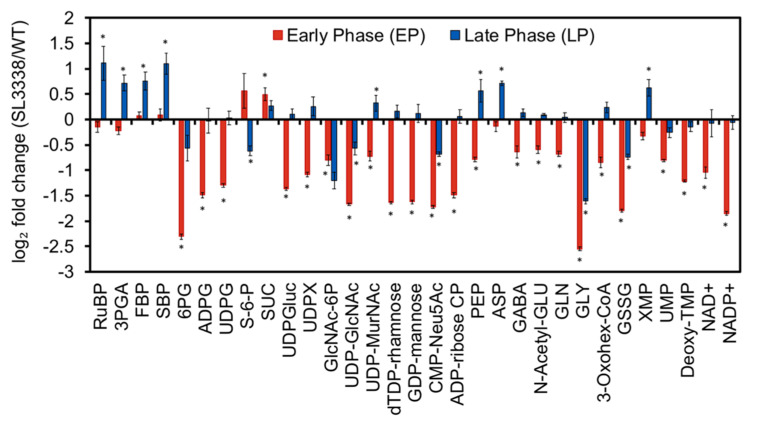
Fold change in the metabolite levels between SL3338 and WT strains at early phase and late phase of growth. While the early phase corresponds to the exponential phase and it was achieved in 18 h, the late phase of growth corresponds to the 96 h when the OD_730nm_ was on average 11. Only those metabolites are included in the panel where the statistically significant fold change was > 1.4 or < 0.71 at *p*-value of < 0.05 in at least one of the conditions. Samples were drawn, quenched and extracted as reported previously [55]. The extracts were mixed with the extract of fully ^13^C-labelled biomass of *S. elongatus* PCC 11801 WT that was used as the internal standard [7]. Fifty metabolites were monitored via LC/MS and quantified as isotopic area ratio. The ratio of the metabolite pool of recombinant to wild type was calculated as ratio of isotopic ratios of recombinant to WT strains and a symbol * represent the metabolites having a fold change of > 1.4 or < 0.71 and a *p*-value of < 0.05 using t-test. The metabolites were identified at MS2 level using the MS-DIAL [61] and MetDIA [62] tool. Error bars shown correspond to SEM from three biological replicates (and three technical replicates for each biological replicate) (n = 3) and are calculated as mentioned in the statistical analysis section. The full list of metabolites with abbreviations is provided in Table S4. More details are presented in Section 3.8.

Transient ^13^C-labelling experiments were performed on both strains by adding ^13^C-labelled sodium bicarbonate to the culture medium during the exponential growth phase [54]. Culture samples were collected at predetermined intervals, quenched, cellular metabolites extracted and ^13^C enrichment quantified via LC/MS/MS [14,54,55,56]. We monitored labelling in 34 metabolites but only those that show satisfactory labelling pattern and appreciable accumulation of ^13^C carbon are shown in Figures S10 and S11. Within the 30-min labelling experiment, a number of intermediate metabolites accumulated substantial amounts of the isotopic ^13^C carbon [57]. On the other hand, the metabolite pool sizes were estimated for both the strains during the early (exponential phase) and late phase of growth. Late phase metabolite profiling was performed to further investigate the possible causes of reduced rate of product formation. As the mass detectors of LC/MS are known to be non-quantitative, relative quantitation of metabolites was obtained via the isotopic ratio method [16]. The metabolite extract from fully ^13^C-labelled biomass of *S. elongatus* PCC 11801 was used as an internal standard, which was mixed with every sample in equal proportion [7]. The monoisotopic ^13^C peak of each metabolite serves as an internal standard thereby accounting for the matrix effects and possible drifts in the MS detector. The ratio of monoisotopic ^12^C and ^13^C peaks for each metabolite thus aids the relative quantitation. Overall, 50 metabolites were monitored (Table S4) but only those that showed fold change of >1.4 or <0.71 at a *p*-value of 0.05 are shown in Figure 6. While the area ratios (^12^C/^13^C) for all the metabolites in wild type and SL3338 during early and late phase are provided in File S2 (Tables S5 and S6), the MID and the mean enrichment values of the metabolites are provided in Files S3 and S4.

#### 2.5.1. Partitioning of Carbon in the Recombinant Strain 

**Figure 7:**
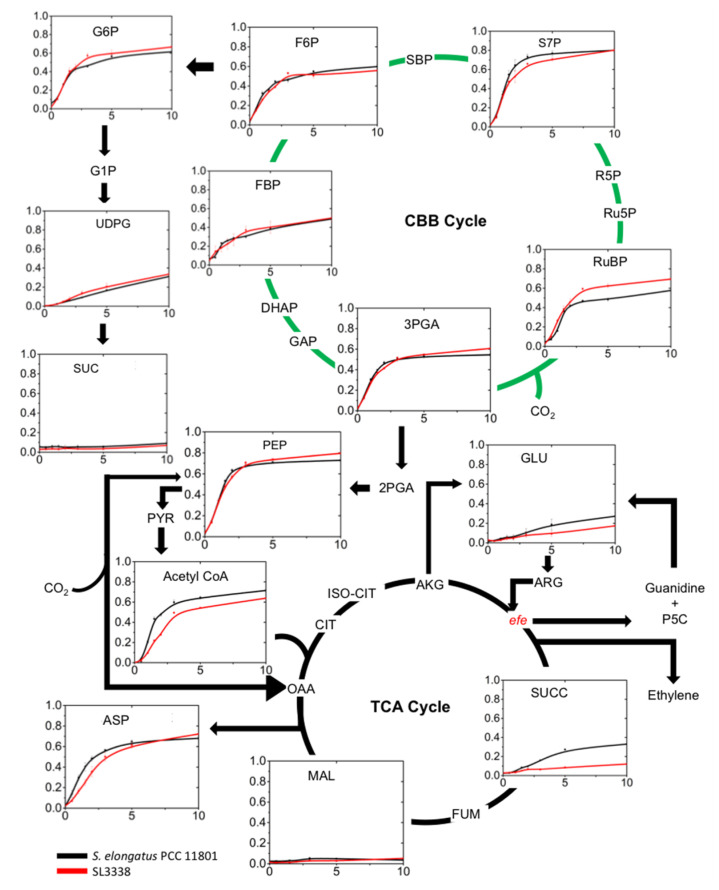
Differences between the WT and SL3338 strains in the ^13^C enrichment of key metabolites in a time-course experiment post addition of ^13^C-sodium bicarbonate. Refer to the legend of Figure 8 and Section 3.9 for experimental details. The values represent the average of two biological replicates (n = 2), each having two technical replicates. Error bars correspond to the SEM of the averaged biological replicates (n = 2). The abbreviations used for metabolites are: Acetyl CoA, acetyl coenzyme A; AKG, alpha-ketoglutaric acid; ARG, arginine; ASP, aspartic acid; CIT, citric acid; DHAP, dihydroxyacetone phosphate; FBP, fructose 1,6 bisphosphate; FUM, dumaric acid; F6P, fructose-6- phosphate; GAP, glyceraldehyde-3-phosphate; GLU, glutamate; G1P, glucose-1-phosphate; G6P, glucose- 6-phosphate; ISO-CIT, isocitric acid; MAL, malic acid; OAA, oxaloacetic acid, PEP, phosphoenolpyruvate; PYR, pyruvic acid; P5C, (S)-1-pyrroline-5-carboxylate; RuBP, ribulose 1,5 bisphosphate; Ru5P, ribulose-5-phosphate; R5P, ribulose-5-phosphate; SBP, sedoheptulose 1,7 bisphosphate; SUC, sucrose; SUCC, succinate; S7P, sedoheptulose-7-bisphosphate; UDPG, UDP-glucose; 2PGA, 2-phosphoglyceric acid; and 3PGA, 3-phosphoglyceric acid. The gene *efe* (ethylene forming enzyme) (red) was inserted into the engineered SL3338 strain.

The Calvin Benson Bassham (CBB) cycle acts as the entry point for the inorganic carbon into the cyanobacterial carbon metabolism. There were no significant differences between the two strains in the pool sizes of the CBB cycle intermediates at early phase (Figure 6). However, the ^13^C labelling studies showed higher ^13^C enrichment at later time points (pseudo isotopic steady state) in the majority of the CBB cycle intermediates with similar initial labelling rates as wild type (Figure 7 and Figure S12). Therefore, the increased enrichment with no significant change in the total pool and rates indicate that the carbon stored as inactive pool of these metabolites may have been utilised for active metabolism in the engineered strain. This is more clearly evident in the plots of the mass isotopologue distribution (MID) of metabolites (Figure S10). The level of M0, the unlabelled state, is expected to reach zero at steady state, which is expected to be seen at 3× the doubling time of the culture. However, in a very short period of 10–30 min, the MIDs of the fast-labelling metabolites reach a pseudo-steady state. To exemplify, the M0 of 3-phosphogyceric acid (3-PGA) reached a pseudo-steady state level of ~ 35% in 10 min (Figure S11). This suggests that there may be an inactive pool of 3-PGA inside the cell, approximately 35% in WT, that does not participate in the active metabolism. In the recombinant strain, the inactive pool of 3-PGA reduces to ~28%. Likewise, we observed a reduction in the inactive pools of ribulose-1,5-bisphosphate (RuBP) and phosphoenolpyruvate (PEP) in the recombinant strain.

**Figure 8:**
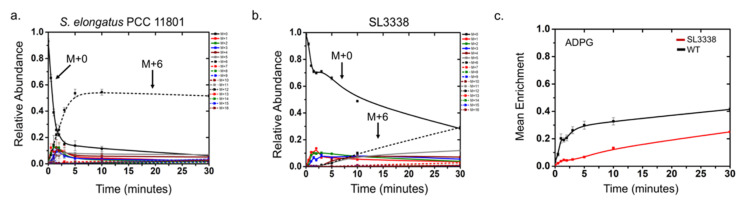
The ^13^C isotopic labelling dynamics of ADP-glucose in the (**a**) WT and (**b**) SL3338 strains. The mass isotopologue distribution (MID) of ADP-Glucose quantified under low light intensity at predetermined intervals after introduction of ^13^C labelled sodium bicarbonate. More details are provided in Section 3.9. (**c**) ^13^C mean enrichment time course of ADP-Glucose for the wild type (black) and recombinant SL3338 (red). The data plotted are the average of two biological replicates (n = 2) with two technical replicates for each biological replicate. Error bars shown correspond to SEM from the averaged biological replicates (n = 2).

Interestingly, despite the absence of obvious differences in the pools of central metabolites, other peripheral metabolites such as sugar nucleotides, which act as activated precursors in the synthesis of storage molecules and cell wall components, were found to be depleted in the engineered strain (Figure 6). This suggests that the cyanobacterial metabolism responds to the stress induced by heterologous pathways by re-partitioning the carbon stored as inactive pools and by lowering the flux towards the synthesis of storage compounds [16,58]. The hypothesis is very well complemented by lower rate of ^13^C enrichment in ADP-glucose, a precursor for glycogen synthesis, and a lower glycogen content (major storage compound in cyanobacteria) in the engineered strain (Figure 7 and Figure 8). On the other hand, the lower abundance of other of metabolites responsible for peptidoglycan biosynthesis (Figure 6) might lead to altered cell wall architecture. Although the cell size was not affected (Figure S13), the cell wall composition can potentially undergo a change in response to the additional metabolic burden. Sucrose acts as an osmolyte and a relatively greater abundance of this metabolite was observed in the engineered strain with a reduced pool of UDP-glucose (UDPG) during the early phase (Figure 6). Moreover, no significant difference in the mean enrichment of UDPG was observed for the first 10 min (Figure 7 and Figure S12). Thus, this increase in the intracellular sucrose pool might be to compensate for the changed osmolarity of the medium due to the presence of extracellular succinate.

Unlike the early phase, the late phase metabolism of the recombinant strain manifested significant accumulation of ribulose bisphosphate (RuBP), 3PGA, fructose-1,6- bisphosphate (FBP) and sedoheptulose-1,7-bisphosphate (SBP) in the recombinant strain (Figure 6). It is noteworthy that the conversion of RuBP, FBP and SBP to respective products is catalysed by the potential flux controlling enzymes, viz., RuBisCO and sedoheptulose/fructose bisphosphatases (SBPase/FBPase) that catalyse irreversible reactions. The accumulation of FBP and SBP suggests that SBPase/FBPase may become limiting during the late phase of metabolism in the engineered strain, as the cells have additional metabolic burden of making products apart from carrying out normal cellular activities. RT-PCR to estimate the mRNA expression levels of SBPase/FBPase showed qualitatively lower expression in recombinant strain (Figure S14). These results suggest that this enzyme may be a major control point and can be overexpressed to improve productivity as observed in earlier reports [7,59,60].

Metabolite profile in late phase shows interesting results. The metabolites that showed significant changes are presented in Figure 6. In contrast to the results for early phase, there was no significant difference in the levels of ADP-Glucose, UDP-Glucose and various other sugar nucleotides (Figure 6) between the engineered and wild type strains. This possibly suggests that wild type and the engineered strain allocates approximately equal carbon towards storage molecules during the late phase metabolism. This is supported by the fact that there is no significant change observed for both glycogen and sucrose between the two strains (Figure 3c, Figure 6). Further, the glycogen content was in general higher in late phase under LL-HC and HL-HC for both the wild type and the engineered strains (Figure 3c and Figure S5). Since the recombinant strain showed increased glycogen content similar to wild type with no change in the relative abundance of ADPG (Figure 3c and Figure 6), knockout of genes for glycogen synthesis might divert more carbon towards the pathway of interest increasing the productivity during the later phase of growth [33].

#### 2.5.2. Lower Part of Glycolysis and the TCA Cycle

The recombinant SL3338 strain showed lower pool of PEP (Figure 6) during the early phase and higher ^13^C enrichment than wild type (Figure 7), suggesting greater carbon flux in the lower branch of glycolysis. However, the lack of difference in pool size and lower rates of ^13^C enrichment of acetyl CoA (Figure 6 and Figure 8) combined with the lower expression of pyruvate dehydrogenase (PDH) (Figure S14) suggest a lower flux through PDH in the recombinant strain. It is likely that a greater proportion of PEP may be diverted to the TCA cycle via PEP carboxylase (*ppc*) in order to replenish the TCA cycle intermediates that get depleted by the action of *efe*. Thus, we suggest simultaneous overexpression of *ppc* and PDH enzymes to improve flux through the TCA cycle. The late phase metabolism showed a higher abundance of PEP and Aspartate (ASP) which may result from slower rates of succinate and ethylene production and higher biomass accumulation (Figure 6). Therefore, overexpression of *ppc* might improve and sustain the productivity by optimising the carbon flow towards TCA cycle [19,22].

Certain metabolites that are directly derived from the intermediates of TCA cycle were observed to be depleted in the early phase suggesting significant product formation (Figure 6). The lower glutamine (GLN) level (Figure 6) depicts more carbon directed to the EFE-catalysed reaction, which utilises 2-oxoglutarate. Since arginine (ARG) is a substrate for ethylene formation and N-acetyl glutamate (N-acetyl GLU) is a precursor for ARG synthesis via the ornithine pathway, the depletion of N-acetyl GLU was not surprising. On the other hand, the abundance of glutamate (GLU) pool was maintained due to a cyclic reaction involving P5C and guanidine which are important for arginine biosynthesis replenishing the GLU pool [40]. Subsequently, the significantly lower rate of ^13^C enrichment for GLU in SL3338 strain than wild type confirmed the efficient partitioning of carbon towards succinate completing the bifurcated TCA cycle due to the expression of *efe* gene (Figure 7). Since the majority of succinate is exported out of the recombinant cell (Figure 2), a basal level of intracellular succinate content was observed similar to the wild type (Figure 6). On the contrary, the low ^13^C mean enrichment pattern observed for succinate in recombinant (Figure 7) might indicate dilution of the intracellular succinate pool due to the rapid exchange of succinate across the leaky cell membrane.

## 3. Materials and Methods 

### 3.1. Chemicals and Reagents

All enzymes were purchased from New England Biolabs (NEB, Ipswich, MA, USA) and ThermoFischer Scientific (Waltham, MA, USA). The isolation and purification kits were obtained from Qiagen (Hilden, Germany). All chemicals, reagents and antibiotics used were of analytical/HPLC grade, whereas reagents for LCMS analysis were of LCMS grade and were procured from Sigma-Aldrich (St. Louis, MO, USA). The *efe* gene [25] was codon optimised for *S. elongatus* PCC 7942 and synthesised from ThermoFischer Scientific (Waltham, MA, USA).

### 3.2. Strain Development Strategy

The markerless, *efe* expressing strain of *S. elongatus* PCC 11801 was developed using the CRISPR-Cpf1 technique as described previously [63]. The protocols in brief is given below.

#### 3.2.1. Plasmid Construction

The pSL2680 vector formed the base plasmid for markerless genome editing [12]. The gRNA sequence was restriction digested and cloned at the *AarI* site of the pSL2680 (Figure S1b) to generate pSL2680-gRNA, which targeted the neutral site I (NSI) sequence of *S. elongatus* PCC 11801. The resultant plasmid pSL2680-gRNA was transformed in *E. coli* XL1-Blue strain and was selected by blue–white screening. Next, the DNA elements comprising of the NSIa, 300 bp cpcB promoter, *efe* gene*-*T*_rrnB_* and NSIb were joined by Gibson Assembly [64] and cloned into the *KpnI* site of the pSL2680-gRNA vector and transformed into *E. coli* XL1-Blue. The resultant plasmid now contains all the elements for genome editing and was named as the editing plasmid pSL3338 (Figure S1b). Due to the small size of the rrnB terminator (T*_rrnB_*), the *efe* gene and the terminator (T*_rrnB_*) were joined separately by overlap PCR before performing Gibson assembly. The NSIa and NSIb correspond to the 750 bp sequences upstream and downstream of the targeting site of gRNA. These plasmids were sequenced from Genewiz^®^ (South Plainfield, NJ). The plasmids were maintained in *E. coli* strain XL1-Blue.

#### 3.2.2. Recombinant Strain Construction via Conjugation

The editing plasmid pSL3338 was transferred in *S. elongatus* PCC 11801 by conjugation as described previously [12]. Briefly, 6 mL of the exponential phase culture of *S. elongatus* PCC 11801 grown in BG11 medium [13], 4 mL culture of the conjugal strain HB101 pRL443 grown in LB with 100 µg/mL ampicillin and 4 mL culture of *E. coli* XL1-Blue strain containing pSL3338 cultivated in LB with 50 µg/mL kanamycin were centrifuged and washed gently. The washed *E. coli* and cyanobacterial cell pellets were mixed together gently in 200 µL of BG-11 medium and evenly spread on to a HATF nitrocellulose filter paper (MilliporeSigma, St. Louis, MO, USA) that was placed on a BG-11 5% LB agar plate without antibiotic, prior to the experiment. The spread plates were incubated for 24 h under 100 µE·m^−2^·s^−1^ light at 38 °C to obtain mat growth of cyanobacterial cells. The filters were then carefully transferred to BG-11 agar plate with 50 µg/mL kanamycin and incubated under same condition until the lawn growth disappeared and individual colonies appeared. The colonies were initially patched on 10 µg/mL kanamycin plates and then further re-streaked on 50 µg/mL kanamycin plate several times (7 streak) to facilitate chromosomal integration and segregation. Segregation was checked by PCR using confirmation primers NS1-CF-F/NSI-CF-R. Post segregation, the recombinant *S. elongatus* PCC11801 strain was cured of the editing plasmid pSL3338 to generate *S. elongatus* PCC 11801:NSI::P*_cpcB_*-*efe*-T*_rrnB_* (referred to as the SL3338 strain) strain by growing in BG11 media without antibiotic pressure, resulting in a clean markerless recombinant strain [63]. All the primers used in the study are listed in Table S1.

### 3.3. Strain Cultivation and Determination of Cell Biomass

The wild type *S. elongatus* PCC 11801 [6] and SL3338 strains were cultivated and maintained in 50 mL BG-11 media at 38 °C under 20 or 300 µE·m^−2^·s^−1^ of light intensity (LL and HL) and ambient air or 1% CO_2_ (LC or HC) in a shaker incubator (Kuhner AG, LT-X, Switzerland). For cultures grown in Multi-Cultivator MC 1000-OD (MC 1000-OD, Photon Systems Instrument, Drásov, Czech Republic), ambient air or air with 1% CO_2_ was bubbled at a rate of 100 sccm per tube. The light intensity varied from 20 to 600 µE·m^−2^·s^−1^ and was specified for each case. The SL3338 as well as wild type cells were grown under different experimental conditions to determine the dry cell weight. Cells were harvested at different time points, washed, dried in flat aluminium cups at 85 °C overnight and their dry weight was measured.

### 3.4. Optimisation of Conditions for Succinate Production 

The SL3338 strain and the wild type *S. elongatus* PCC 11801 were grown under different conditions in shaker incubator and MC 1000-OD to optimise the succinate titre. The effect of carbon in sufficient and limited states on succinate production was tested in shaker incubator under 200 µE·m^−2^·s^−1^ light intensity with or without 50 mM bicarbonate. The light characterisation was performed under four different light intensities of 20, 50, 100 and 300 µE·m^−2^·s^−1^ in MC 1000-OD bubbled with 1% CO_2_. The effect of O_2_ was investigated by cultivating the strains in different growth vessels (baffled flask, unbaffled flask and bottle) under optimal light and CO_2_ levels for succinate production. The starting inoculum was maintained around 0.1–0.2 OD_730nm_. For estimating succinate levels in the extracellular media, 1 m of culture was collected every day from each experimental setup, centrifuged and the supernatant was used for measuring the succinate titre using the succinate estimation kit from Megazyme (Bray, Ireland). The assay was performed according to the manufacturers’ instructions. Samples were temporarily stored at −20 °C until ready for analysis. All experiments were performed in triplicates.

### 3.5. Effect of Nitrate Limitation on Succinate Production

The wild type and the recombinant cells were inoculated in 25 mL shake flask with an OD_730nm_ of 0.2 in 7 mL BG-11_0_ media supplemented with three different concentration of NaNO_3_, 1.5 (termed as 1× or as present in the BG-11 medium), 0.75 (0.5×) and 0.375 g/L (0.25×) in shaker incubator at 120, under 20 µE·m^−2^·s^−1^ light and 1% CO_2_ to study the effect of nitrate concentration on succinate production.

### 3.6. Estimation of Ethylene Production Using GC-FID

The recombinant and wild type *S. elongatus* PCC 11801 strains were grown under optimal growth conditions in shake flask or multicultivator and transferred to 120 mL air-tight rubber cock Hungate glass bottles to measure ethylene formation rates. The initial OD_730nm_ was adjusted to 0.1, 0.2 or 2.15 as specified and the total culture volume was adjusted to 20 mL using BG-11 media. The bottles were incubated in shaker incubator under 20 µE·m^−2^·s^−1^ light intensity. Ethylene accumulated in the head space of the culturing bottle was estimated using GC-FID (6890N Gas Chromatograph-Flame Ionization Detector, Agilent). Four hundred microlitres of gas from the head space of the bottle was sampled every hour using an air-tight syringe and was injected in GC-FID. An ethylene standard curve was prepared prior the experimentation for quantitative estimation of ethylene. The gas samples were separated in a Poropak N column having inner dimensions of 5′ × 1/8″ and detected by flame ionisation detector using argon as the carrier gas at a flow rate of 65 ml/min. The oven temperature was maintained at 100 °C, whereas the injector and detector temperatures were kept at 150 and 200 °C, respectively [65].

### 3.7. Isolation of Total RNA and Reverse Transcriptase (RT)-PCR of Selected Genes

RNA isolation kit from Sigma-Aldrich (St. Louis, MO, USA) was used for RNA extraction from cell pellets. The isolated total RNA was cleaned to remove genomic DNA contamination and then proceeded to convert the total RNA to cDNA by iScript Advanced cDNA synthesis kit from Bio-Rad Laboratories (Hercules, CA, USA) by following the manufacturer’s instructions. Subsequently, the comparative transcript levels of the genes were determined by qualitative RT-PCR with 30 PCR cycles. The respective genes were amplified from the cDNA using respective set of primers (Table S1) and visualised on a 0.8% agarose gel.

### 3.8. Metabolite Pool Size Estimation of SL3338 Strain

*S. elongatus* PCC 11801 WT and SL3338 were grown under 1% CO_2_ and 20 µE·m^−2^·s^−1^ in CO_2_ incubator shaker at 38 °C. Metabolomic analysis was performed with cells that were at exponential growth phase (OD_730nm_ of 0.6) and stationary phase (4th day, OD_730nm_ of ~11). Metabolite extraction was performed as described previously [55]. Briefly, the sample volume was adjusted to use 3 mg DCW. The culture was filtered rapidly through nylon membrane filters (Whatman, 0.8 µ) in the presence of light. To quench the metabolism, the biomass together with the filter was transferred quickly to 80:20 methanol:water, which was precooled to −80 °C. The metabolites were extracted as described [14,55] and the extracts were lyophilised and stored at −80 °C until ready for LCMS analysis. The metabolite extracts were reconstituted in 100 μL of water:methanol mixture (50/50, *v*/*v*) and filtered. Then 20 µL of the reconstituted metabolite extract was mixed with equal volume (20 µL) of metabolite extract fully ^13^C-labelled biomass of *S. elongatus* PCC 11801, which served as the internal standard for every metabolite. Eight microlitres of the sample was injected on LCMS. The data were acquired using information dependent acquisition (IDA) method on a Q-TOF instrument (TripleTOF 5600, SCIEX, Framingham, MA) interfaced with Shimadzu Ultra Performance-Liquid Chromatography (UPLC) system (Shimadzu, Nexera LC −30 AD, Singapore). The instrument was operated under negative ion mode. Reverse phase ion-pairing chromatography was performed using C18 Synergi 4 μm Hydro-RP LC column 150 × 2 mm (Phenomenex Inc, Torrance, CA, USA). The gradient program and other instrument parameters were set as reported previously [14]. The relative quantification of metabolites was performed by normalising area under the peak for monoisotopic m/z of a particular metabolite by its respective ^13^C-monoisotopic isotopologue present in the internal standard giving isotopic area ratio. The areas were estimated using using Multiquant 3.0.2 (Sciex, Framingham, MA, USA). The fold change of metabolite pools between the SL3338 and WT strains was calculated by dividing the isotopic area ratios (i.e., ^12^C/^13^C) of a metabolite in SL3338 by that of WT. These experiments were performed in three independent biological replicates with three technical replicates for each experiment (n = 3).

### 3.9. ^13^C Enrichment Analysis of SL3338 Strain

The ^13^C labelling experiment was conducted as described previously [54] under ambient air and 20 µE·m^−2^·s^−1^ light intensity in an incubator shaker at 38 °C in a shake flask. Briefly, the labelling experiment was performed when the culture reached exponential growth phase (0.6–0.7 OD_730nm_). Twenty millilitre sample corresponding to the time (t = 0) (unlabelled) was collected. Then, the cultures were cultivated for 30 min to avoid dilution by unlabelled CO_2_ into the system by restricting the exchange of air. Post incubation, 1g/L ^13^C-Sodium bicarbonate was added to the system and 20 mL of culture were rapidly sampled at various timepoints (0, 0.5, 1, 1.5, 2, 3, 5, 10 and 30 min). The culture samples were filtered rapidly through a 0.8 µm Whatman nylon membrane filter (Catalogue No. 7408-004) procured from Merck Millipore (MilliporeSigma, St. Louis, MO, USA) in the presence of light and then quenched quickly by submerging the biomass together with the filter paper in chilled 80:20 methanol:water (precooled at −80 °C).

The extraction of metabolite and LCMS analysis was done as described in Section 3.8. The extracted ion chromatograms (XIC) of metabolites were visualised with PeakView 2.2 software and MasterView1.0 (Sciex, Framingham, MA, USA). The areas under the curve for the XICs corresponding to each isotopologue for each metabolite were estimated using Multiquant 3.0.2 (Sciex, Framingham, MA, USA) and then converted to mass isotopologue distribution (MID). All the experiments were performed in two independent biological replicates and two technical replicates for each set, n = 2. MIDs for each technical replicate were then corrected for natural abundance of the ^13^C isotope using the IsoCor software, as described by Millard et al. [66,67]. Average values obtained and statistical analysis performed on IsoCor corrected data.

### 3.10. Determination of Photosynthetic Efficiency and Capacity

The maximal PSII quantum yield (F_V_/F_M_) and the PSII photosynthetic capacity (ETRmax) was determined for the wild type and recombinant SL3338 strains in vivo by using a pulse amplitude modulated fluorimeter (Phyto-PAM, Heinz Walz, Germany). Briefly, 3 mL of exponentially grown culture (under optimal production condition in shake flask) was dark-adapted for 15–20 min before measurement under the same experimental growth temperature. The F_0_, Fm, Fv/Fm and ETRmax were determined with the saturation pulse method and Rapid Light Curve as per the instruction manual and previous reports [68,69].

### 3.11. Statistical Analysis

Statistical significance of the product titres and the metabolite levels between the engineered and wild type strains were examined by t-test. Metabolites with a fold change of ≥1.4 or ≤0.71 between the engineered and WT strains and *p*-value < 0.05 were considered as statistically significant.

The standard error of mean (SEM) of ratio of metabolite levels between the engineered and WT strains, denoted as *X* and *Y*, respectively, was calculated as follows. The variance of the ratio *X*/*Y* was first calculated using Taylor expansion method according to Equation (3) [70]:

(3) $$\;\sigma^{2}{}_{\frac{X}{Y}}=\frac{1}{{\bar{Y}}^{2}}\sigma_{X}^{2}-2\frac{\bar{X}}{{\bar{Y}}^{3}}\;cov\;\left(X,Y\right)+\frac{{\bar{X}}^{2}}{{\bar{Y}}^{4}}\;\sigma_{Y}^{2}$$

The fold change between two conditions is represented as:

(4) $$\;\frac{\bar{X}}{\bar{Y}}\;\pm \;\frac{\sigma_{\frac{X}{Y}}}{\sqrt{n}}$$

where *σ* denotes the standard deviation and *σ*^2^ is the variance amd *X* and *Y* represents two conditions. *X* is engineered strain, whereas *Y* is wild type strain. $\bar{X}$ and $\bar{Y}$ are the respective mean of the metabolite levels in the *X* and *Y* conditions and cov(*X*,*Y*) implies the sample covariance. The number of replicates is denoted by *n*.

## 4. Conclusions

Metabolic engineering of cyanobacteria for the co-production of two essential products in two different phases might be economically advantageous. To that end, the EFE enzyme was expressed under a strong P_cpcB7942_ promoter in a fast-growing cyanobacterium, *S. elongatus* PCC 11801, to simultaneously produce ethylene and succinate. The best production condition was observed to be low light, high CO_2_, and this might be due to the enhanced activity of the promoter in use [41] or the activated TCA cycle [43]. The low light production strategy in MC 1000-OD ensured the accumulation of lower biomass with higher cumulative product titre. On the contrary, cultivation in shake flask limited the carbon partitioning towards the product of interest. Under optimal conditions, SL3338 strain co-produced succinate and ethylene at peak specific production rates of 338.26 and 1044.18 μmole/gDCW/h, respectively. These results obtained by expressing a single copy of the *efe* gene in *S. elongatus* PCC 11801 compare favourably with the reported productivities for strains that have been engineered either with multiple copies of *efe* or have additional genetic modifications. Further, metabolite profiling was performed to identify the potential bottlenecks under low light and 1% CO_2_ in the shake flask. The metabolome analysis showed that the recombinant SL3338 strain during the early phase did not alter the relative pool sizes of CBB cycle intermediates but enhanced ^13^C enrichment at later time points (pseudo isotopic steady state) towards the CBB metabolites. This suggests better utilisation of the inactive pools of CBB cycle intermediate, in turn improving the carbon capture efficiency of the cycle in recombinant strain. Since the glycogen accumulation was lower in engineered strain than the wild type, the increased carbon uptake was diverted towards the product of interest. A similar result was observed for the *efe*-expressing *Synechocystis* sp. Strain, which also showed improved flux diverted from the carbon fixation cycle towards ethylene production [30]. Though the recombinant strain increased the efficiency of carbon sequestration, SBPase/FBPase enzyme remains to be a major control point in the pathway. Beside SBPase, PDH and ppc enzymes have also been identified as the bottlenecks. As future work, tuning the potential metabolic control knobs identified in this study might help enhance the photosynthetic co-production of ethylene and succinate.

## Figures and Tables

**Table 1:** Comparison of reported specific productivity of succinate and ethylene in cyanobacteria with the values obtained in this study.

SL. No.	Year	Organisms	Genetic Modifications	Peak Ethylene Productivity (µmole/g/h)	Peak Succinate Productivity (mg/g/h)	Ref.
1	2013	*Synechocystis* sp. PCC 6803	*2X efe*	44	---	[25]
2	2015	*Synechocystis* sp. PCC 6803	*1X efe*, modified RBS	128.21	---	[30]
3	2015	*Synechocystis* sp. PCC 6803	*	86.61	---	[31]
4	2015	*Synechocystis* sp. PCC 6803	∆*ackA* and O.E.(*SigE*)	---	0.2	[32]
5	2016	*S. elongatus* PCC 7942	O.E.(*gabD*/*kgd*/*ppc*/*gltA*)	---	10.67	[19]
6	2017	*Synechocystis* sp. PCC 6803	#	439	---	[26]
7	2017	*Synechocystis* sp. PCC 6803	slr0168:: Ptrc-Rbs30-*efe*-Km^r^	15.82	---	[33]
8	2018	*Synechocystis* sp. PCC 6803	O.E.(*ppc*) and ∆*ackA*	---	1.0	[22]
9	2019	*S. elongatus* PCC 7942	NSI:: Ptrc- *efe*-TrrnB	25	---	[24]
10	2020	*Synechocystis* sp. PCC 6803	PnrsB-*efe*, O.E.(2X*ppc*, *ppsA_7002_*)	118.84	---	[29]
11		*S. elongatus* PCC 11801	NSI::P_cpcB300_-*efe*-T_rrnB_	338.26	121.1	This study

* slr0168::Sp^r^-PcpcB-*efe*/sll1981::Km^r^-PcpcB-*efe*/slr0370::Cm^r^-PcpcB-*efe*/phaAB::Gm^r^-pcpcB-*kgtP*; # slr0168::Sp^r^-PcpcB-*efe*/sll1981::Km^r^-PcpcB-*efe*/slr0370::Cm^r^-PcpcB-*efe*/sll1423::Gm^r^-pcpcB-*efe.*

## Supplementary Materials

The following are available online at https://www.mdpi.com/2218-1989/10/6/250/s1, 1. Supplementary Information containing figures and tables: Figure S1: The plasmid map for markerless genome editing of cyanobacterial genome using CRISPR-Cpf1: (a) the schematic showing the construct assembled using Gibson Assembly along with the respective primers; and (b) pSL3338; Figure S2: The Genome and RNA level confirmation; Figure S3: The biomass accumulation of the wild type and recombinant strains; Figure S4: The effect aeration on succinate levels was studied by growing the strain in different cultivation vessels: air-tight bottle, baffled shake flask and unbaffled shake flask; Figure S5: Estimation of glycogen accumulated in wild type and recombinant SL3338 strains under high light and 1% CO_2_ in shaker incubator; Figure S6: Estimation of succinate levels and growth profile of recombinant SL3338 strain grown in MC 1000-OD at 1% CO_2_ bubbled under high light intensity; Figure S7: The comparison of the growth profile (representative) of *S. elongatus* PCC 11801 when the culture was cultivated in BG11 media with or without buffered pH; Figure S8: Re-using biomass for ethylene production over a period of three days; Figure S9: The carbon partitioning between biomass and product of interest when cultivated in bottle; Figure S10: The mass isotopologoue distribution of the metabolites under study in wild type and the recombinant strains; Figure S11: The comparison of mass isotopologue of metabolites under study quantified from *S. elongatus* PCC 11801 and recombinant strain; Figure S12: Comparison of mean enrichment of metabolites between the wild type and recombinant strains after: (a) 10 min; and (b) 30 min; Figure S13: The microscopic image of the wild type and recombinant strains; Figure S14: Qualitative RT-PCR was performed for both the wild type and recombinant strains; Table S1: The list of primers employed in the study; Table S2: Detailed comparison of succinate and ethylene productivity reported in the literature for different cyanobacteria; Table S3: The Quantum efficiency (Fv/Fm) of the wild type and recombinant strain; Table S4: List of metabolites analysed in the study. 2. File S2: Table S5: The area ratios (^12^C/^13^C) for all the metabolites in the wild type and SL3338 strain of *S. elongatus* PCC 11801 under early phase (EP) (XLSX); Table S6: The area ratios (^12^C/^13^C) for all the metabolites in the wild type and SL3338 strain of *S. elongatu*s PCC 11801 under late phase (EP) (XLSX). 3. File S3: Mass isotopologue distributions (MIDs) of the metabolites (under study) observed in wild type *S. elongatus* PCC 11801 and SL3338 (XLSX). 4. File S4: Mass enrichment of the metabolites (under study) observed in wild type *S. elongatus* PCC 11801 and SL3338 (XLSX).

## References

[1] Nozzi N.E., Oliver J.W.K., Atsumi S. (2013). Cyanobacteria as a Platform for Biofuel Production. Front. Bioeng. Biotechnol..

[2] Mukherjee B., Madhu S., Wangikar P.P. (2020). The role of systems biology in developing non-model cyanobacteria as hosts for chemical production. Curr. Opin. Biotechnol..

[3] Sengupta A., Pakrasi B. H., Wangikar P. P. (2018). Recent advances in synthetic biology of cyanobacteria. Appl. Microbiol. Biotechnol..

[4] Santos-Merino M., Singh A.K., Ducat D.C. (2019). New Applications of Synthetic Biology Tools for Cyanobacterial Metabolic Engineering. Front. Bioeng. Biotechnol..

[5] Yu J., Liberton M., Cliften P.F., Head R.D., Jacobs J.M., Smith R.D., Koppenaal D.W., Brand J.J., Pakrasi H.B. (2015). *Synechococcus elongatus* UTEX 2973, a fast growing cyanobacterial chassis for biosynthesis using light and CO₂. Sci. Rep..

[6] Jaiswal D., Sengupta A., Sohoni S., Sengupta S., Phadnavis A.G., Pakrasi H.B., Wangikar P.P. (2018). Genome Features and Biochemical Characteristics of a Robust, Fast Growing and Naturally Transformable Cyanobacterium *Synechococcus elongatus* PCC 11801 Isolated from India. Sci. Rep..

[7] Jaiswal D., Sengupta A., Sengupta S., Madhu S., Pakrasi H.B., Wangikar P.P. (2020). A Novel Cyanobacterium *Synechococcus elongatus* PCC 11802 has Distinct Genomic and Metabolomic Characteristics Compared to its Neighbor PCC 11801. Sci. Rep..

[8] Włodarczyk A., Selão T.T., Norling B., Nixon P.J. (2020). Newly discovered *Synechococcus* sp. PCC 11901 is a robust cyanobacterial strain for high biomass production. Commun. Biol..

[9] Vasudevan R., Gale G.A.R., Schiavon A.A., Puzorjov A., Malin J., Gillespie M.D., Vavitsas K., Zulkower V., Wang B., Howe C.J. (2019). CyanoGate: A Modular Cloning Suite for Engineering Cyanobacteria Based on the Plant MoClo Syntax. Plant Physiol..

[10] Knoot C.J., Khatri Y., Hohlman R.M., Sherman D.H., Pakrasi H.B. (2019). Engineered Production of Hapalindole Alkaloids in the Cyanobacterium *Synechococcus* sp. UTEX 2973. ACS Synth. Biol..

[11] Li S., Sun T., Xu C., Chen L., Zhang W. (2018). Development and optimization of genetic toolboxes for a fast-growing cyanobacterium *Synechococcus elongatus* UTEX 2973. Metab. Eng..

[12] Ungerer J., Pakrasi H.B. (2016). Cpf1 Is A Versatile Tool for CRISPR Genome Editing Across Diverse Species of Cyanobacteria. Sci. Rep..

[13] Wendt K.E., Ungerer J., Cobb R.E., Zhao H., Pakrasi H.B. (2016). CRISPR/Cas9 mediated targeted mutagenesis of the fast growing cyanobacterium *Synechococcus elongatus* UTEX 2973. Microb. Cell Fact..

[14] Jaiswal D., Prasannan C.B., Hendry J.I., Wangikar P.P. (2018). SWATH Tandem Mass Spectrometry Workflow for Quantification of Mass Isotopologue Distribution of Intracellular Metabolites and Fragments Labeled with Isotopic ^13^C Carbon. Anal. Chem..

[15] Dange M.C., Mishra V., Mukherjee B., Jaiswal D., Merchant M.S., Prasannan C.B., Wangikar P.P. (2020). Evaluation of freely available software tools for untargeted quantification of ^13^C isotopic enrichment in cellular metabolome from HR-LC/MS data. Metab. Eng. Commun..

[16] Abernathy M.H., Yu J., Ma F., Liberton M., Ungerer J., Hollinshead W.D., Gopalakrishnan S., He L., Maranas C.D., Pakrasi H.B. (2017). Deciphering cyanobacterial phenotypes for fast photoautotrophic growth via isotopically nonstationary metabolic flux analysis. Biotechnol. Biofuels.

[17] Knoot C.J., Ungerer J., Wangikar P.P., Pakrasi H.B. (2018). Cyanobacteria: Promising biocatalysts for sustainable chemical production. J. Biol. Chem..

[18] Zhang S., Bryant D.A. (2011). The tricarboxylic acid cycle in cyanobacteria. Science.

[19] Lan E.I., Wei C.T. (2016). Metabolic engineering of cyanobacteria for the photosynthetic production of succinate. Metab. Eng..

[20] Li H., Shen C.R., Huang C., Sung L., Wu M., Hu Y. (2016). CRISPR-Cas9 for the genome engineering of cyanobacteria and succinate production. Metab. Eng..

[21] Huang C.H., Shen C.R., Li H., Sung L.Y., Wu M.Y., Hu Y.C. (2016). CRISPR interference (CRISPRi) for gene regulation and succinate production in cyanobacterium *S. elongatus* PCC 7942. Microb. Cell Fact..

[22] Hasunuma T., Matsuda M., Kato Y., Vavricka C.J., Kondo A. (2018). Temperature enhanced succinate production concurrent with increased central metabolism turnover in the cyanobacterium *Synechocystis* sp. PCC 6803. Metab. Eng..

[23] Shabestary K., Anfelt J., Ljungqvist E., Jahn M., Yao L., Hudson E.P. (2018). Targeted Repression of Essential Genes to Arrest Growth and Increase Carbon Partitioning and Biofuel Titers in Cyanobacteria. ACS Synth. Biol..

[24] Carbonell V., Vuorio E., Aro E.M., Kallio P. (2019). Enhanced stable production of ethylene in photosynthetic cyanobacterium *Synechococcus elongatus* PCC 7942. World J. Microbiol. Biotechnol..

[25] Ungerer J., Tao L., Davis M., Ghirardi M., Maness P.C., Yu J. (2012). Sustained photosynthetic conversion of CO2 to ethylene in recombinant cyanobacterium *Synechocystis* 6803. Energy Environ. Sci..

[26] Mo H., Xie X., Zhu T., Lu X. (2017). Effects of global transcription factor NtcA on photosynthetic production of ethylene in recombinant *Synechocystis* sp. PCC 6803. Biotechnol. Biofuels.

[27] Eckert C., Xu W., Xiong W., Lynch S., Ungerer J., Tao L., Gill R., Maness P.-C., Yu J. (2014). Ethylene-forming enzyme and bioethylene production. Biotechnol. Biofuels.

[28] Wang B., Dong T., Myrlie A., Gu L., Zhu H., Xiong W., Maness P., Zhou R., Yu J. (2019). Photosynthetic production of the nitrogen-rich compound guanidine. Green Chem..

[29] Durall C., Lindberg P., Yu J., Lindblad P. (2020). Increased ethylene production by overexpressing phosphoenolpyruvate carboxylase in the cyanobacterium *Synechocystis* PCC 6803. Biotechnol. Biofuels.

[30] Xiong W., Morgan J.A., Ungerer J., Wang B., Maness P.-C., Yu J. (2015). The plasticity of cyanobacterial metabolism supports direct CO_2_ conversion to ethylene. Nat. Plants.

[31] Zhu T., Xie X., Li Z., Tan X., Lu X. (2015). Enhancing photosynthetic production of ethylene in genetically engineered *Synechocystis* sp. PCC 6803. Green Chem..

[32] Osanai T., Shirai T., Iijima H., Nakaya Y., Okamoto M., Kondo A., Hirai M.Y. (2015). Genetic manipulation of a metabolic enzyme and a transcriptional regulator increasing succinate excretion from unicellular cyanobacterium. Front. Microbiol..

[33] Veetil V.P., Angermayr S.A., Hellingwerf K.J. (2017). Ethylene production with engineered *Synechocystis* sp PCC 6803 strains. Microb. Cell Fact..

[34] Hagemann M., Hess W.R. (2018). Systems and synthetic biology for the biotechnological application of cyanobacteria. Curr. Opin. Biotechnol..

[35] Fell D.A. (1998). Increasing the flux in metabolic pathways: A metabolic control analysis perspective. Biotechnol. Bioeng..

[36] Cheah Y.E., Xu Y., Sacco S.A., Babele P.K., Zheng A.O., Johnson C.H., Young J.D. (2020). Systematic identification and elimination of flux bottlenecks in the aldehyde production pathway of *Synechococcus elongatus* PCC 7942. Metab. Eng..

[37] McAtee A.G., Jazmin L.J., Young J.D. (2015). Application of isotope labeling experiments and ^13^C flux analysis to enable rational pathway engineering. Curr. Opin. Biotechnol..

[38] Nagahama K., Ogawa T., Fujii T., Tazaki M., Tanase S., Morino Y., Fukuuda H. (1991). Purification and Properties of an ethylene-forming enzyme from *Pseudomonas syringae* pv. phaseolicola PK2. J. Gen. Microbiol..

[39] Fukuda H., Takahira O., Masato T., Kazuhiro N., Takao F., Sumio T., Yoshimasa M. (1992). Two reactions are simultaneously catalyzed by a single enzyme: The arginine-dependent simultaneous formation of two products, ethylene and succinate, from 2-oxoglutarate by an enzyme from *Pseudomonas syringae*. Biochem. Biophys. Res. Commun..

[40] Knoop H., Steuer R. (2015). A Computational Analysis of Stoichiometric Constraints and Trade-Offs in Cyanobacterial Biofuel Production. Front. Bioeng. Biotechnol..

[41] Sengupta A., Sunder A.V., Sohoni S.V., Wangikar P.P. (2019). Fine-Tuning Native Promoters of *Synechococcus elongatus* PCC 7942 to Develop a Synthetic Toolbox for Heterologous Protein Expression. ACS Synth. Biol..

[42] Asplund-Samuelsson J., Janasch M., Hudson E.P. (2018). Thermodynamic analysis of computed pathways integrated into the metabolic networks of *E. coli* and *Synechocystis* reveals contrasting expansion potential. Metab. Eng..

[43] Xiong W., Brune D., Vermaas W.F.J. (2014). The γ-aminobutyric acid shunt contributes to closing the tricarboxylic acid cycle in *Synechocystis* sp. PCC 6803. Mol. Microbiol..

[44] Stockel J., Welsh E.A., Liberton M., Kunnvakkam R., Aurora R., Pakrasi H.B. (2008). Global transcriptomic analysis of *Cyanothece* 51142 reveals robust diurnal oscillation of central metabolic processes. Proc. Natl. Acad. Sci. USA.

[45] Hewett J.P., Wolfe A.K., Bergmann R.A., Stelling S.C., Davis K.L. (2016). Human health and environmental risks posed by synthetic biology R D for energy applications: A literature analysis. J. ABSA Int..

[46] Yen U.C., Huang T.C., Yen T.C. (2004). Observation of the circadian photosynthetic rhythm in cyanobacteria with a dissolved-oxygen meter. Plant. Sci..

[47] Sengupta A., Wangikar P.P. (2020). A method to compute instantaneous oxygen evolution rates in cyanobacterial cultures grown in shake flasks. Eng. Reports.

[48] Gründel M., Scheunemann R., Lockau W., Zilliges Y. (2012). Impaired glycogen synthesis causes metabolic overflow reactions and affects stress responses in the cyanobacterium *Synechocystis* sp. PCC 6803. Microbiology.

[49] Salar-García M.J., Bernal V., Pastor J.M., Salvador M., Argandoña M., Nieto J.J., Vargas C., Cánovas M. (2017). Understanding the interplay of carbon and nitrogen supply for ectoines production and metabolic overflow in high density cultures of *Chromohalobacter salexigens*. Microb. Cell Fact..

[50] Yao L., Cengic I., Anfelt J., Hudson E.P. (2016). Multiple Gene Repression in Cyanobacteria Using CRISPRi. ACS Synth. Biol..

[51] Díaz-Troya S., Roldán M., Mallén-Ponce M.J., Ortega-Martínez P., Florencio F.J. (2019). Lethality caused by ADP-glucose accumulation is suppressed by salt-induced carbon flux redirection in cyanobacteria. J. Exp. Bot..

[52] Cano M., Holland S.C., Artier J., Burnap R.L., Ghirardi M., Morgan J.A., Yu J. (2018). Glycogen Synthesis and Metabolite Overflow Contribute to Energy Balancing in Cyanobacteria. Cell Rep..

[53] Visser D., Heijnen J.J. (2002). The mathematics of Metabolic Control Analysis revisited. Metab. Eng..

[54] Hendry J.I., Prasannan C., Ma F., Möllers K.B., Jaiswal D., Digmurti M., Allen D.K., Frigaard N.U., Dasgupta S., Wangikar P.P. (2017). Rerouting of carbon flux in a glycogen mutant of cyanobacteria assessed via isotopically non-stationary ^13^C metabolic flux analysis. Biotechnol. Bioeng..

[55] Prasannan C.B., Jaiswal D., Davis R., Wangikar P.P. (2018). An improved method for extraction of polar and charged metabolites from cyanobacteria. PLoS ONE.

[56] Prasannan C.B., Mishra V., Jaiswal D., Wangikar P.P. (2019). Mass Isotopologue Distribution of dimer ion adducts of intracellular metabolites for potential applications in ^13^C Metabolic Flux Analysis. PLoS ONE.

[57] Nöh K., Grönke K., Luo B., Takors R., Oldiges M., Wiechert W. (2007). Metabolic flux analysis at ultra short time scale: Isotopically non-stationary ^13^C labeling experiments. J. Biotechnol..

[58] Abernathy M.H., Czajka J.J., Allen D.K., Hill N.C., Jeffrey C., Tang Y.J. (2019). Cyanobacterial carboxysome mutant analysis reveals the influence of enzyme compartmentalization on cellular metabolism and metabolic network rigidity. Metab. Eng..

[59] Janasch M., Asplund-Samuelsson J., Steuer R., Hudson E.P. (2019). Kinetic modeling of the Calvin cycle identifies flux control and stable metabolomes in *Synechocystis* carbon fixation. J. Exp. Bot..

[60] Liang F., Lindberg P., Lindblad P. (2018). Engineering photoautotrophic carbon fixation for enhanced growth and productivity. Sustain. Energy Fuels.

[61] Tsugawa H., Cajka T., Kind T., Ma Y., Higgins B., Ikeda K., Kanazawa M., VanderGheynst J., Fiehn O., Arita M. (2015). MS-DIAL: Data-independent MS/MS deconvolution for comprehensive metabolome analysis. Nat. Methods.

[62] Li H., Cai Y., Guo Y., Chen F., Zhu Z.J. (2016). MetDIA: Targeted Metabolite Extraction of Multiplexed MS/MS Spectra Generated by Data-Independent Acquisition. Anal. Chem..

[63] Ungerer J., Wendt K.E., Hendry J.I., Maranas C.D., Pakrasi H.B. (2018). Comparative genomics reveals the molecular determinants of rapid growth of the cyanobacterium *Synechococcus elongatus* UTEX 2973. Proc. Natl. Acad. Sci. USA.

[64] Gibson D.G., Young L., Chuang R.-Y., Venter J.C., Hutchison III C.A., Smith H.O. (2009). Enzymatic assembly of DNA molecules up to several hundred kilobases. Nat. Methods.

[65] Bandyopadhyay A., Stöckel J., Min H., Sherman L.A., Pakrasi H.B. (2010). High rates of photobiological H_2_ production by a cyanobacterium under aerobic conditions. Nat. Commun..

[66] Millard P., Delépine B., Guionnet M., Heuillet M., Bellvert F., Létisse F. (2019). IsoCor: Isotope correction for high-resolution MS labeling experiments. Bioinformatics.

[67] Millard P., Letisse F., Sokol S., Portais J.C. (2012). IsoCor: Correcting MS data in isotope labeling experiments. Bioinformatics.

[68] Varshney P., Beardall J., Bhattacharya S., Wangikar P.P. (2020). Effect of elevated carbon dioxide and nitric oxide on the physiological responses of two green algae, *Asterarcys quadricellulare* and *Chlorella sorokiniana*. J. Appl. Phycol..

[69] Pierangelini M., Stojkovic S., Orr P.T., Beardall J. (2014). Photosynthetic characteristics of two *Cylindrospermopsis raciborskii* strains differing in their toxicity. J. Phycol..

[70] Wolter K.M. (2007). Taylor Series Methods. Introduction to Variance Estimation.

