# Supplementary material for: Photosynthetic Co-production of Succinate and Ethylene in a Fast-Growing Cyanobacterium, Synechococcus elongatus PCC 11801
Source: Metabolites. 2020 Jun 16;10(6):250. doi: 10.3390/metabo10060250 (PMC7345232; doi:10.3390/metabo10060250)
Supplement: Supplementary file 1 [file metabolites-10-00250-s001.zip › Supplementary Files-R3/Supplementary information.pdf]

Supplementary Information

Photosynthetic co-production of succinate and ethylene in a fast-growing cyanobacterium, *Synechococcus elongatus* PCC 11801

Annesha Sengupta [1], Prem Pritam [1#], Damini Jaiswal [1#], Anindita Bandyopadhyay [2], Himadri B. Pakrasi [2,3,*], and Pramod P. Wangikar [1,4,5,*]

[1]Department of Chemical Engineering, Indian Institute of Technology Bombay, Powai, Mumbai 400076 India.
[2]Department of Biology, Washington University, St. Louis, Missouri, USA
[3]Department of Energy, Environmental and Chemical Engineering, Washington University, St. Louis, Missouri, USA.
[4]DBT-Pan IIT Center for Bioenergy, Indian Institute of Technology Bombay, Powai, Mumbai 400076 India.
[5]Wadhwani Research Center for Bioengineering, Indian Institute of Technology Bombay, Powai, Mumbai 400076 India.

#Contributed equally

*Correspondence: wangikar@iitb.ac.in; Tel: +91 22 25767232; Fax: + 91 22 25726895 (P.P.W). pakrasi@wustl.edu ; Ph: 314-935-6853 (H.B.P).

a.

b.

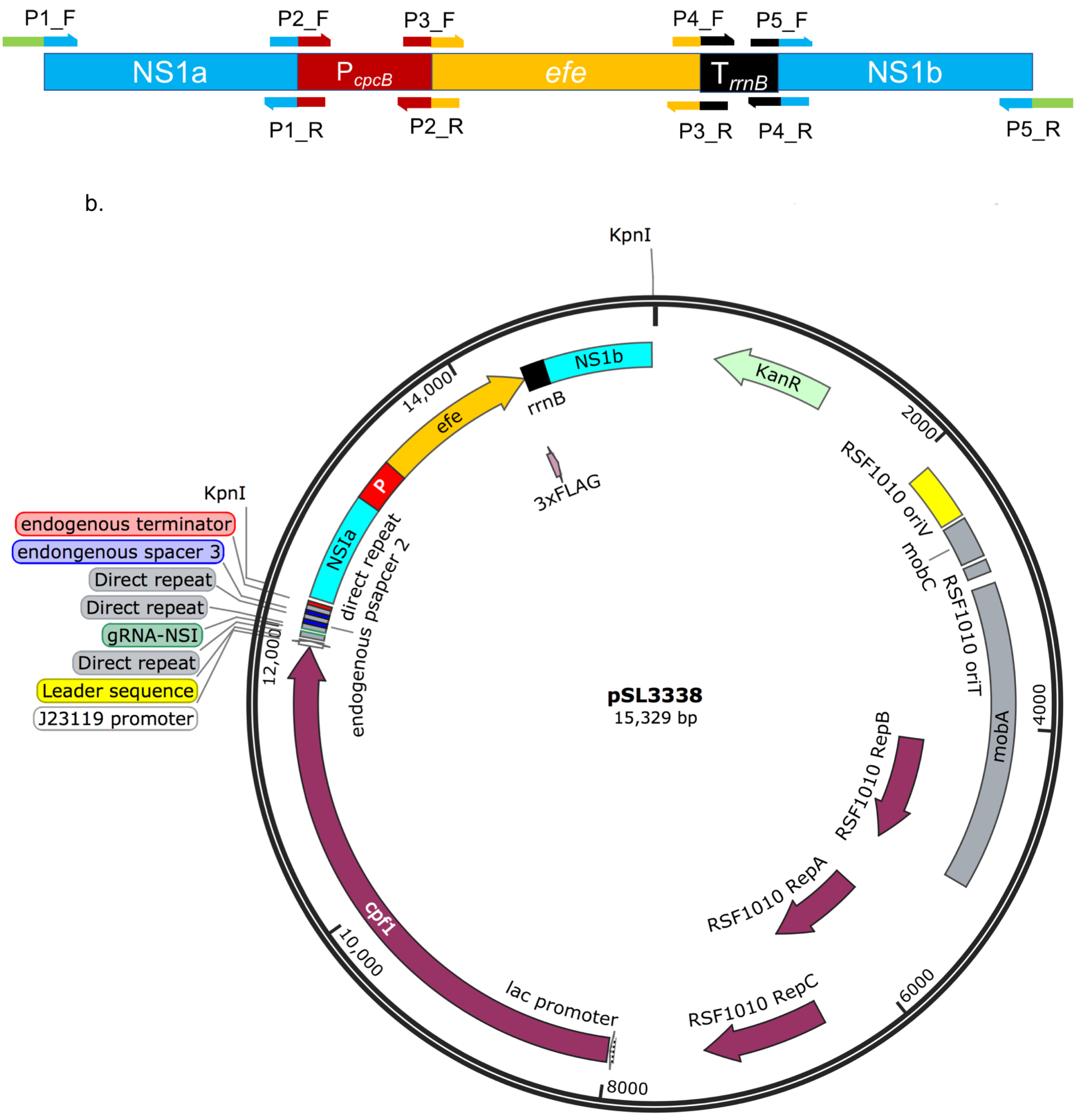

Figure S1: The plasmid map for markerless genome editing of cyanobacterial genome using CRISPR-Cpf1. (a) The schematic showing the construct assembled using Gibson Assembly along with the respective primers. The primers are represented by single-head arrow and the tail of the arrow signifies the overhang required to join different gene fragments. The colour of the primers are represented by the same colour as the genetic element to be amplified and so as the overhang in the primers. The green overhang in primer P1 and P5 represent the overhangs corresponding to the plasmid backbone in order to ligate with the CRISPR plasmid containing the gRNA at the AarI site. (b) The pSL3338 plasmid containing the gRNA targeting the NSI site of the *S. elongatus* PCC 11801 genome (gRNA-NSI) at the AarI RE site and the EFE-construct (NS1a-P_{cpcB}-*efe*-T_{rrnB}-NS1b) at the KpnI site. Ethylene forming enzyme (*efe*).

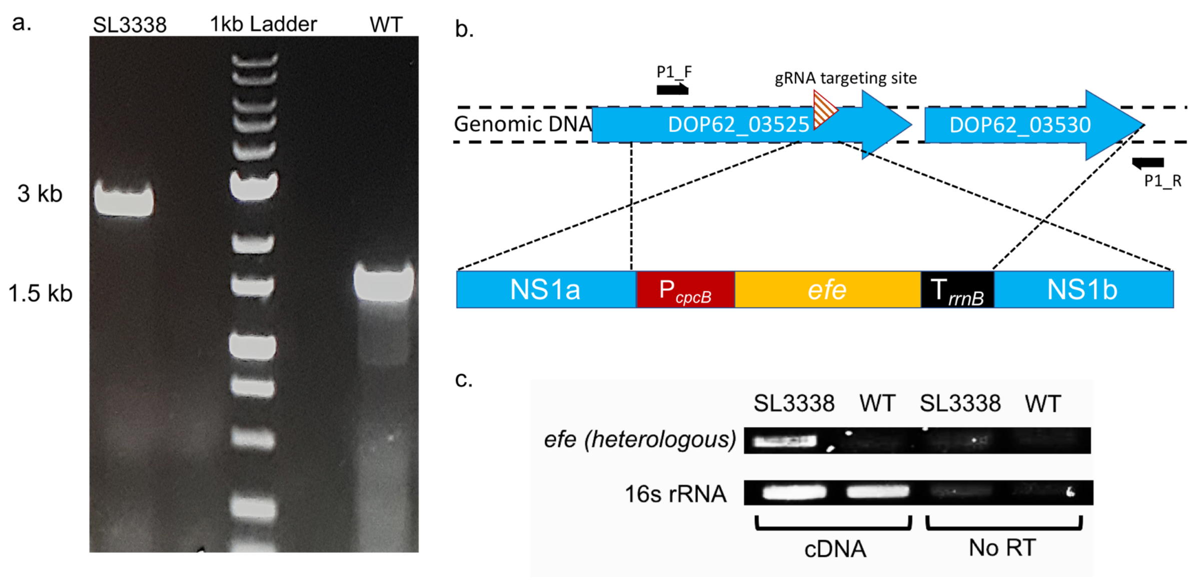

Figure S2: The Genome and RNA level confirmation: (a) the agarose gel picture showing the segregated band of heterologous ethylene forming enzyme (*efe*) gene integrated at the neutral site (NS) 1 of *S. elongatus* PCC 11801 genome. The 1.5 kb band corresponds to the intact NSI site of the wild type genome (b) The scheme showing the genomic arrangement of *S. elongatus* PCC 11801 where the construct has been inserted in to the genome. P1 represent the primers used for the genomic confirmation (c) The mRNA level of the *efe* gene observed for the recombinant strain as compared to the wild type strain. 16S rRNA primers were used as a loading control. *S. elongatus* PCC 11801: WT and *S. elongatus* PCC 11801-NSI::P_{cpcB}-*efe*-T_{rrnB}: SL3338

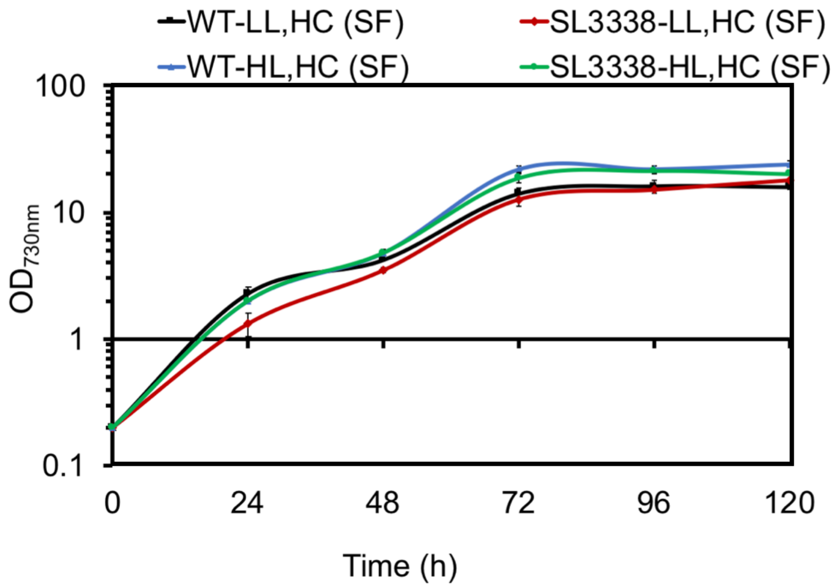

Figure S3: The biomass accumulation of the wild type and recombinant strains as a function of OD at 730nm over a period of 5 days in shake flask (SF) under HC and LL/HL. Error bars correspond to SEM of the biological replicates (n=3). *S. elongatus* PCC 11801: WT and *S. elongatus* PCC 11801-NSI::P_{cpcB}-*efe*-T_{rrnB}: SL3338

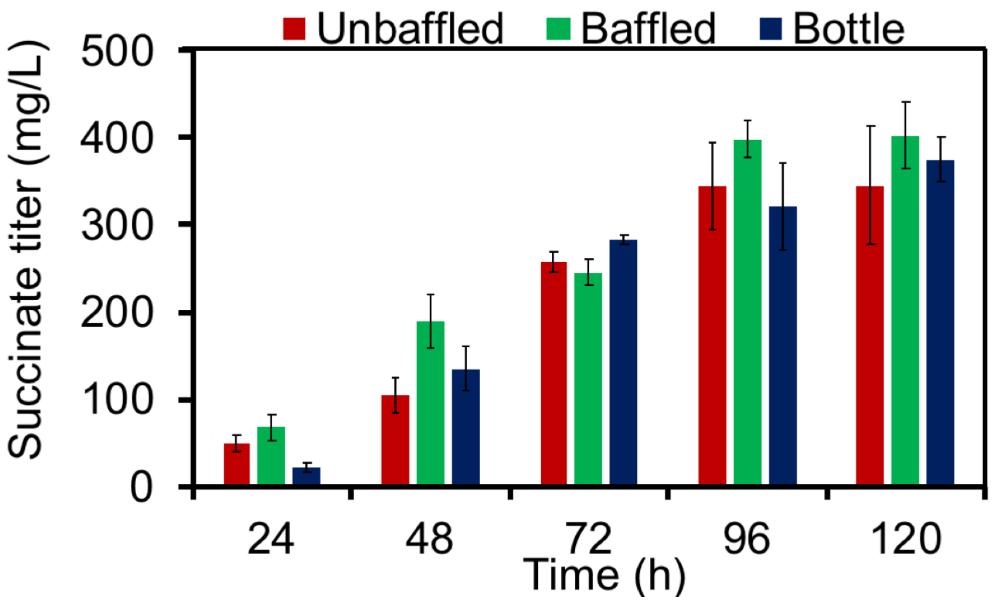

Figure S4: The effect aeration on succinate levels was studied by growing the SL3338 strain in different cultivation vessels, air-tight bottle, baffled shake flask and unbaffled shake flask under 20 μE.m^{-2}.s^{-1} light in shake incubator with an agitation of 120 rpm. The experiment was done under saturated carbon levels. While for flasks 1% CO_2 was used, 50 mM bicarbonate was supplemented to the medium every 24 h in the bottle. Error bars correspond to SEM of the biological replicates (n=3).

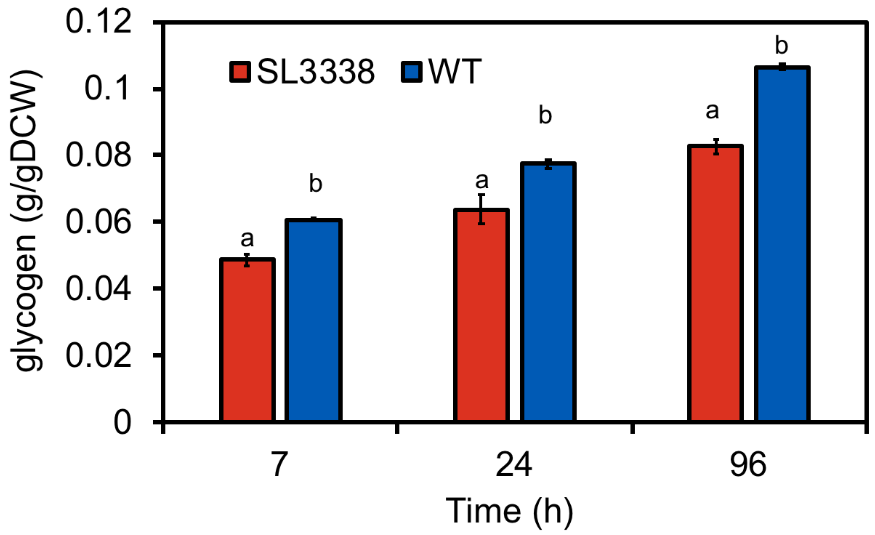

Figure S5: Estimation of glycogen accumulated in wild type and recombinant SL3338 strains under high light (300 μE.m^{-2}.s^{-1}) and 1% CO_2 in shaker incubator. *S. elongatus* PCC 11801: WT and *S. elongatus* PCC 11801-NSI::P_{cpcB}-*efe*-T_{rrnB}: SL3338. The error bars correspond to SEM from three biological and two technical replicates (n=3) and the letters a and b denote statistically different values of μ for each category ($P < 0.05$) using T-test.

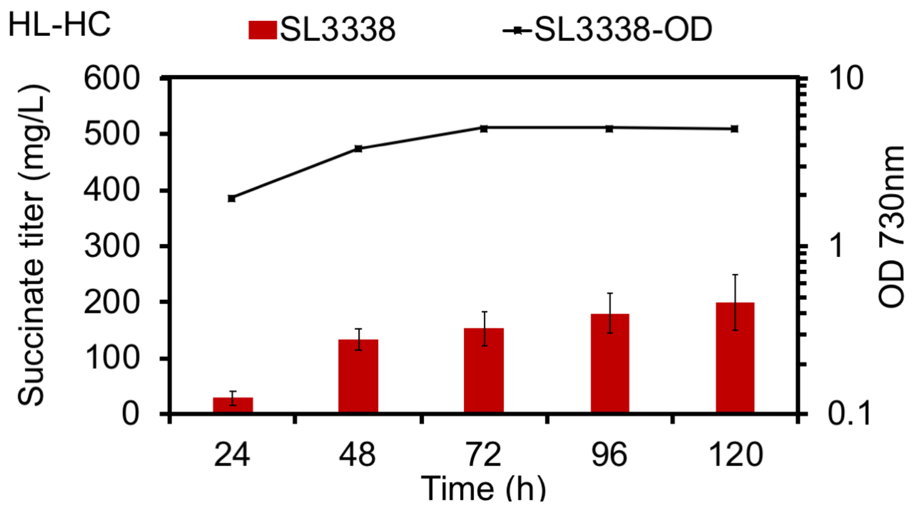

Figure S6: Estimation of succinate levels and growth profile of recombinant SL3338 strain grown in MC 1000-OD at 1% CO_2 bubbled under high light intensity of 300 µE.m^{-2}.s^{-1}. Error bars correspond to SEM of the biological replicates (n=3). SL3338: *S. elongatus* PCC 11801-NSI::P$_{cpcB}$-*efe*-T$_{rrnB}$.

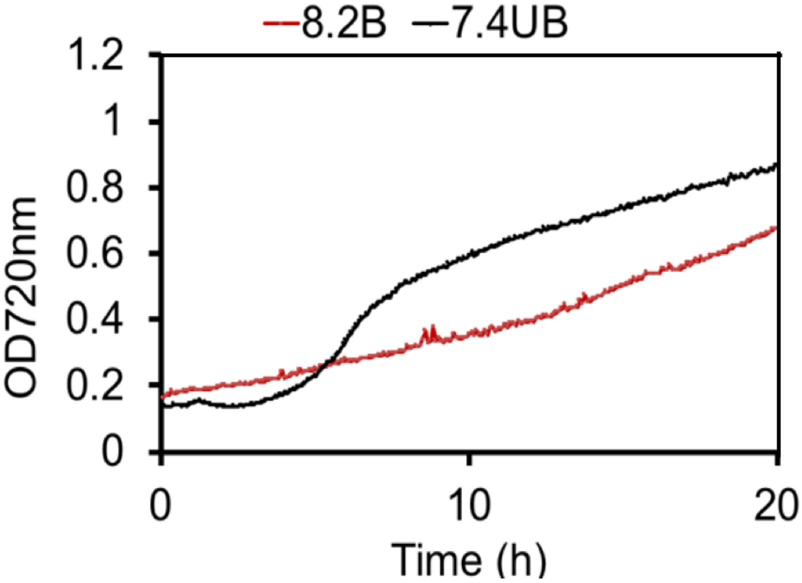

Figure S7: The comparison of the growth profile (representative) of *S. elongatus* PCC 11801 when the culture was cultivated in BG11 media with or without buffered pH. The experiment was conducted in MC 1000-OD under ambient condition, 1000 μE.m^{-2}.s^{-1} and 42 ˚C. While 8.2B indicate buffered BG11 media where the pH is maintained at 8.2 over the run using Tes-KOH (8.2), 7.4UB indicate unbuffered BG11 media where the initial media pH is set at 7.4 but during the run the pH is not maintained.

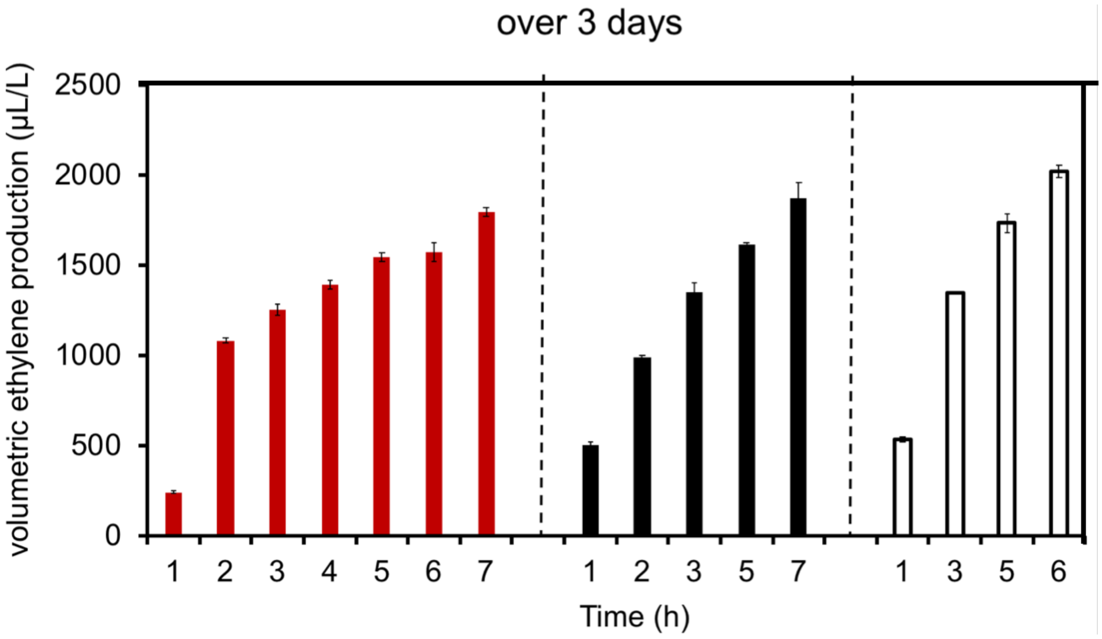

Figure S8: The biomass used for ethylene production can be re-used for at least three days without compromising its volumetric productivity. The SL3338 cells were initially grown under high light intensity and 1% CO_2 to an exponential OD_{730nm}, which was used as an inoculum to incubate for ethylene production. An OD of 0.2 was incubated in air-tight bottle for 8 h and ethylene was measured every hr using GC-FID. Post 8h of incubation, these cells were re-inoculated in shake flask and grown for 16h under high light and CO_2 condition. These growing cells were then again used for ethylene measurement under low light condition on the 2nd day. Same procedure was repeated for the 3rd day. Error bars correspond to SEM of the biological replicates (n=3). SL3338: *S. elongatus* PCC 11801-NSI::P_{cpcB}-*efe*-T_{rrnB}

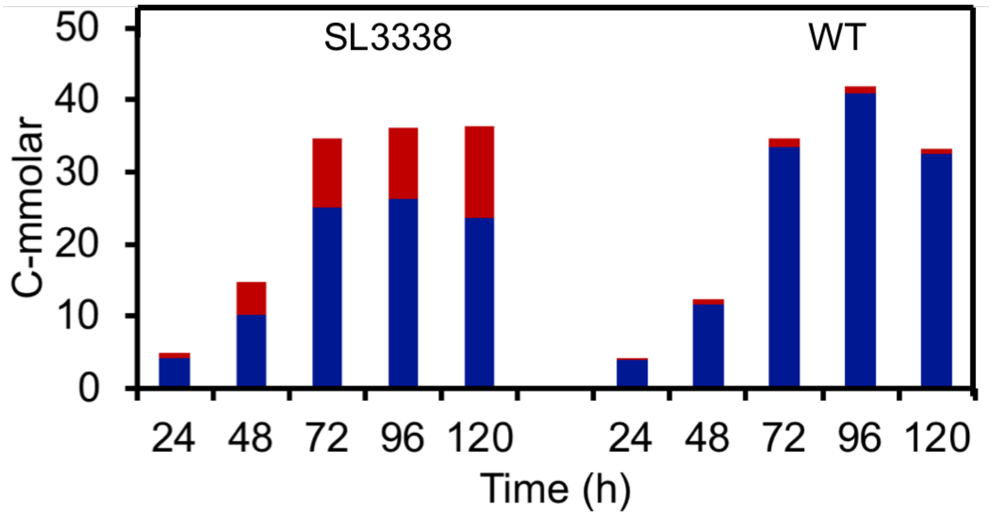

Figure S9: The carbon partitioning between biomass and product of interest when SL3338 strain was cultivated in bottle with BG11 supplemented with 50 mM bicarbonate under 20 μE.m^{-2}.s^{-1}.

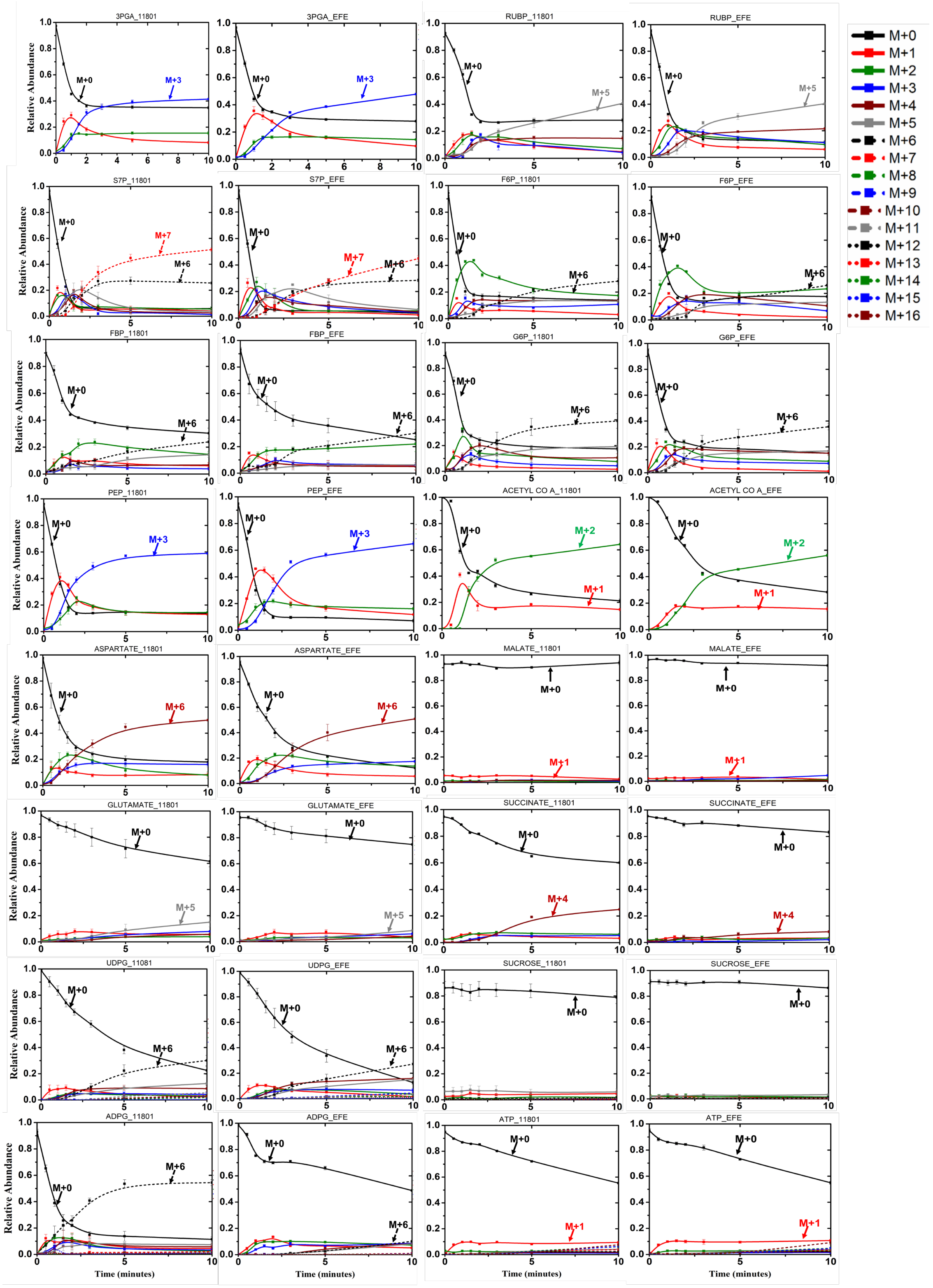

Figure S10: The mass isotopologoue distribution of the metabolites under study in wild type (11801) and the recombinant *S. elongatus* PCC 11801-NSI::P$_{cpcB}$-*efe*-T$_{rrnB}$ (EFE) strains. The data plotted is an average of two biological replicates (n=2) with two technical replicates for each biological replicate. Error bars shown correspond to SEM from the averaged biological replicates. The abbreviations used for metabolites are Acetyl CoA: acetyl coenzyme A; ADPG: ADP-glucose; ATP: Adenosine triphosphate; FBP: fructose 1,6 bisphosphate; F6P: fructose-6- phosphate; G6P: glucose- 6-phosphate; PEP: phosphoenolpyruvate; RuBP: ribulose 1,5 bisphosphate; S7P: sedoheptulose-7-bisphosphate; UDPG: UDP-glucose; 2PGA: 2-phosphoglyceric acid; and 3PGA: 3-phosphoglyceric acid.

RUBP

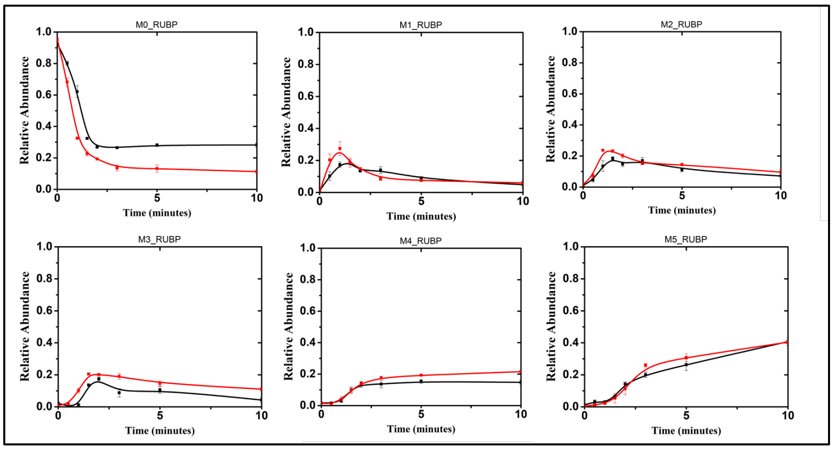

3-PGA

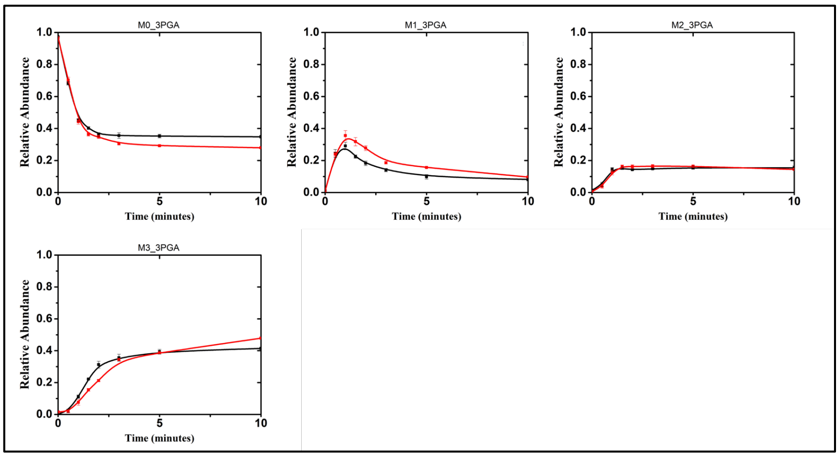

FBP

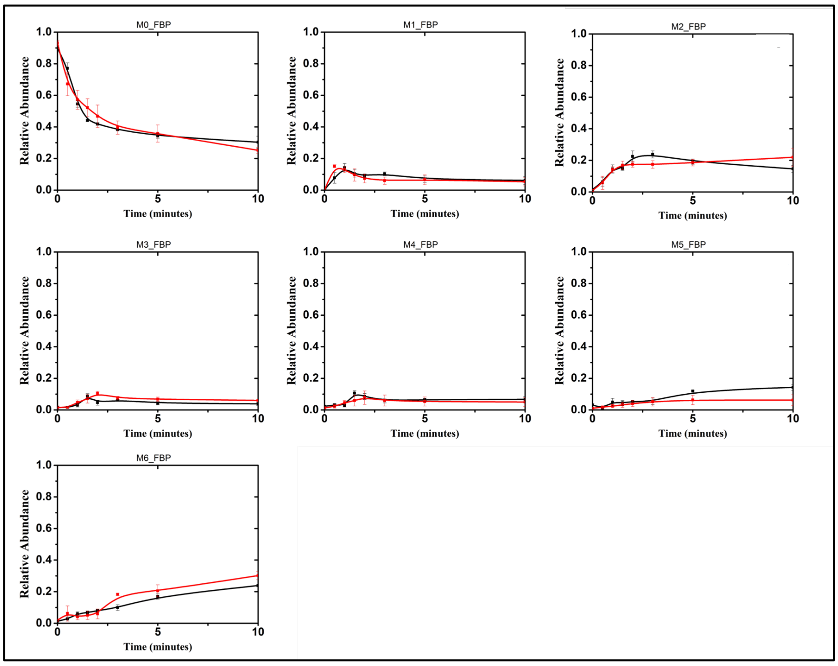

F6P

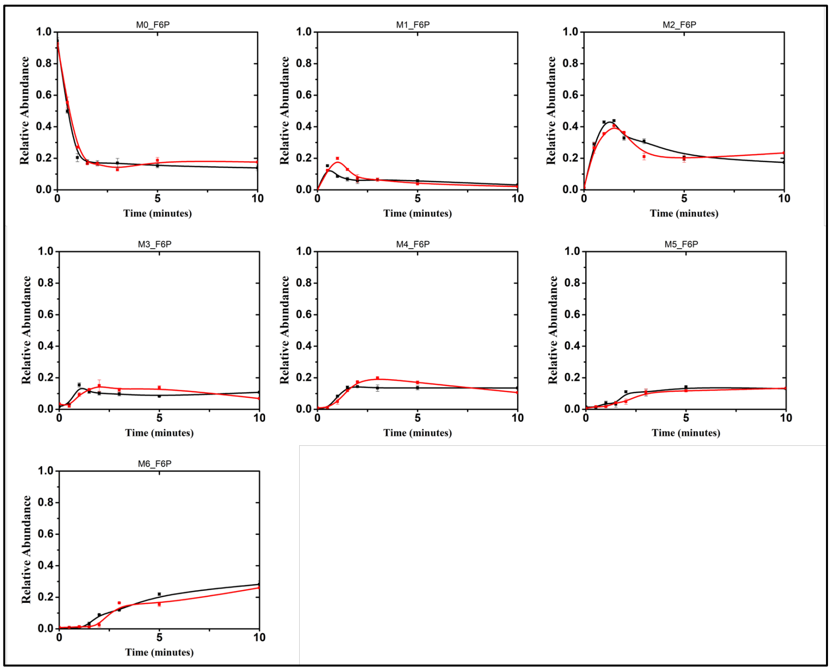
M0_F6P
M1_F6P
M2_F6P
M3_F6P
M4_F6P
M5_F6P
M6_F6P
Relative Abundance
Time (minutes)

S7P

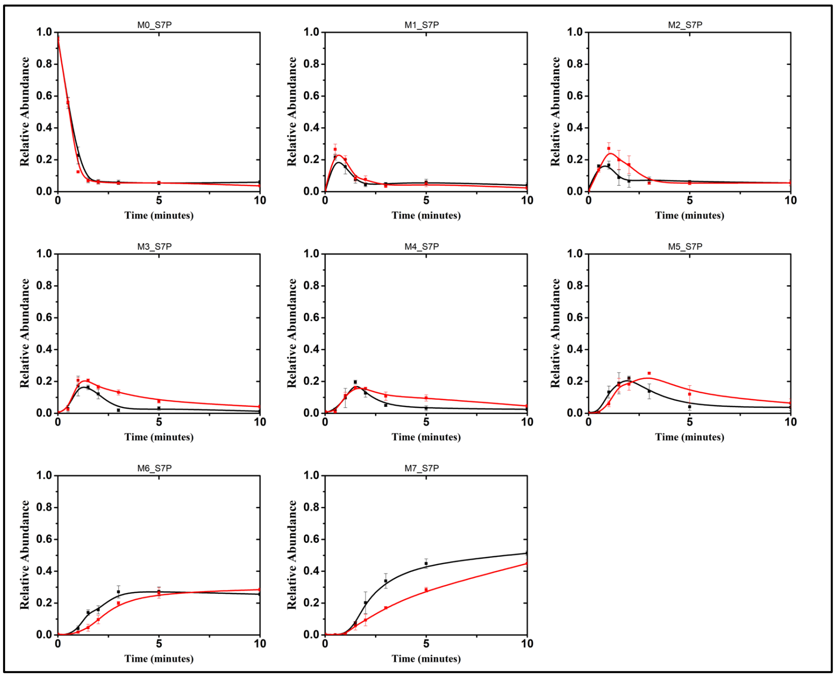
M0_S7P
M1_S7P
M2_S7P
M3_S7P
M4_S7P
M5_S7P
M6_S7P
M7_S7P
Relative Abundance
Time (minutes)

G6P

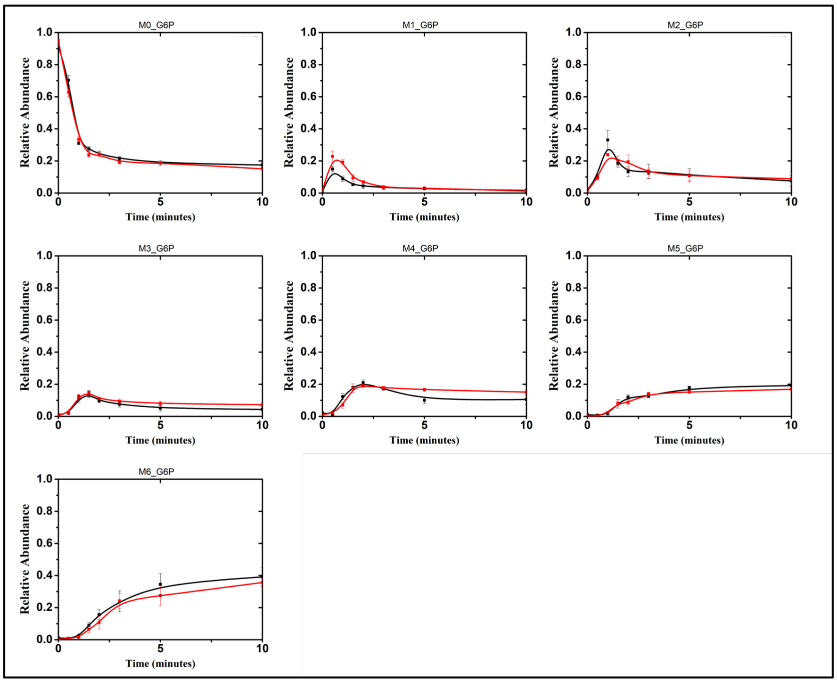
M0_G6P
M1_G6P
M2_G6P
M3_G6P
M4_G6P
M5_G6P
M6_G6P
Relative Abundance
Time (minutes)
0.0
0.2
0.4
0.6
0.8
1.0
0
5
10

ADPG

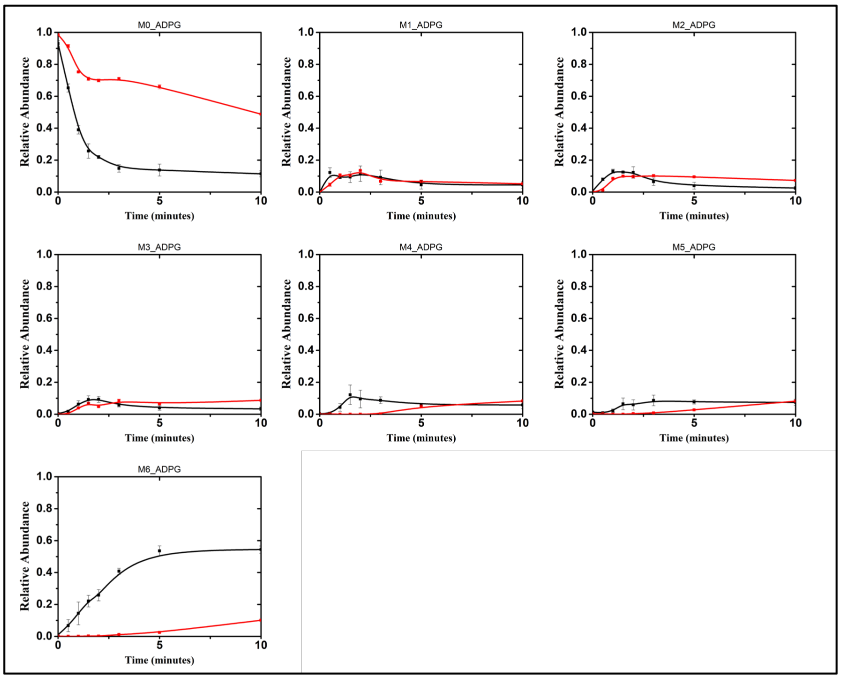
M0_ADPG
M1_ADPG
M2_ADPG
M3_ADPG
M4_ADPG
M5_ADPG
M6_ADPG
Relative Abundance
Time (minutes)
0.0
0.2
0.4
0.6
0.8
1.0
0
5
10

UDPG

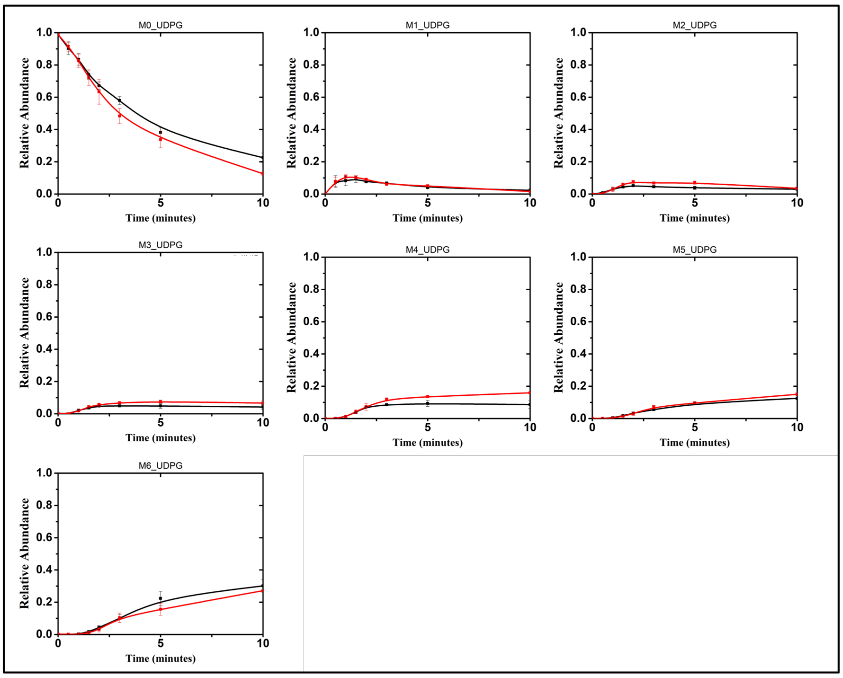

PEP

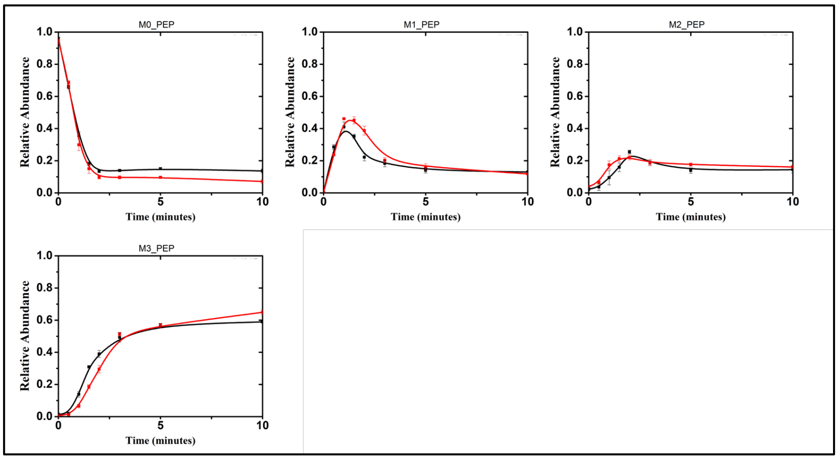

ACETYL COA

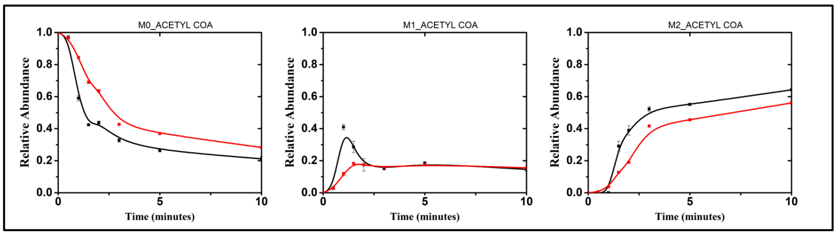

ASPARTATE

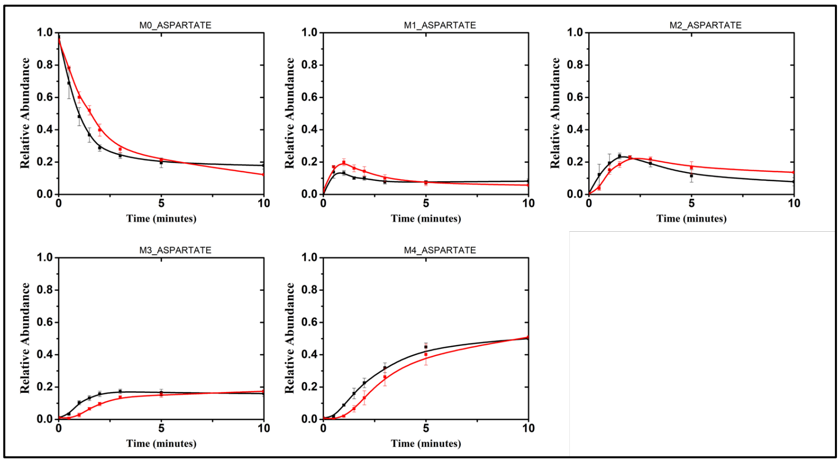

SUCROSE

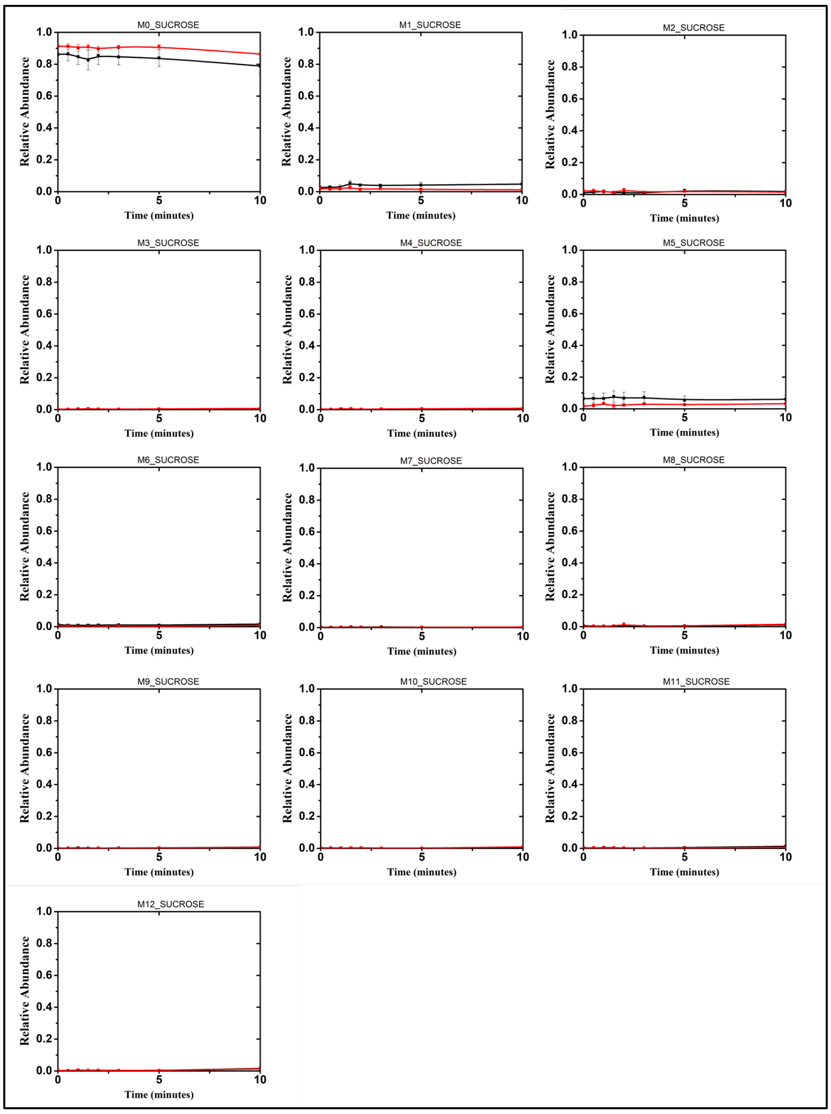

MALATE

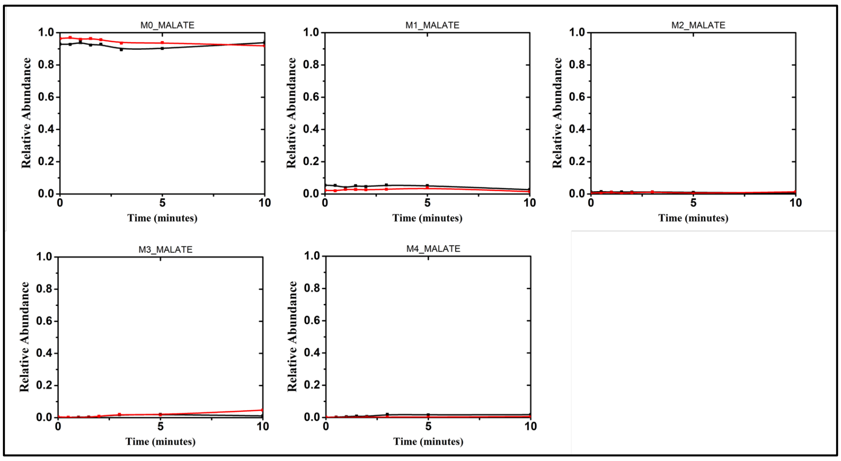

SUCCINATE

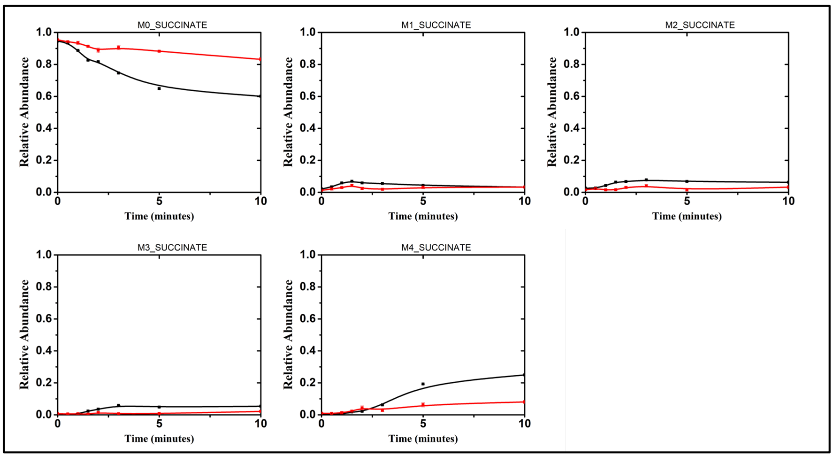

GLUTAMATE

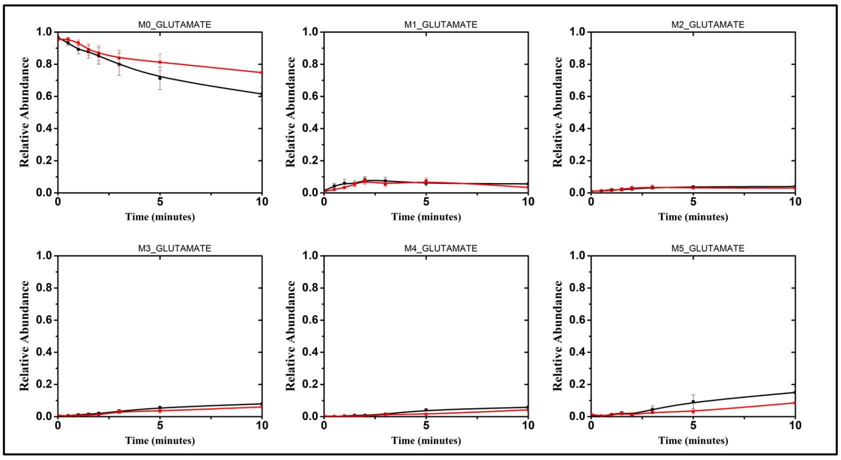

ATP

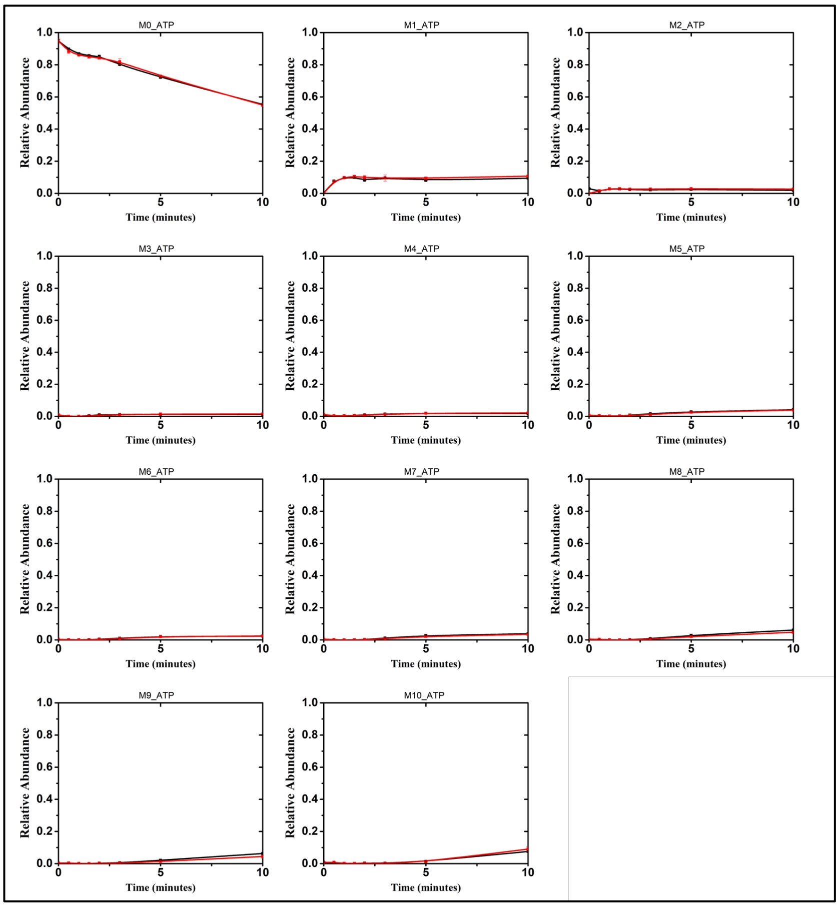

Figure S11: The comparison of mass isotopologue of metabolites under study quantified from *S. elongatus* PCC 11801 (black trace) and recombinant *S. elongatus* PCC 11801-NSI::P_{cpcB}-*efe*-T_{rrnB} strain (red trace). The data plotted is an average of two biological replicates (n=2) with two technical replicates for each biological replicate. Error bars shown correspond to SEM from the averaged biological replicates. The abbreviations used for metabolites are Acetyl CoA: acetyl coenzyme A; ADPG: ADP-glucose; ATP: Adenosine triphosphate; FBP: fructose 1,6 bisphosphate; F6P: fructose-6- phosphate; G6P: glucose- 6-phosphate; PEP: phosphoenolpyruvate; RuBP: ribulose 1,5 bisphosphate; S7P: sedoheptulose-7-bisphosphate; UDPG: UDP-glucose; 2PGA: 2-phosphoglyceric acid; and 3PGA: 3-phosphoglyceric acid.

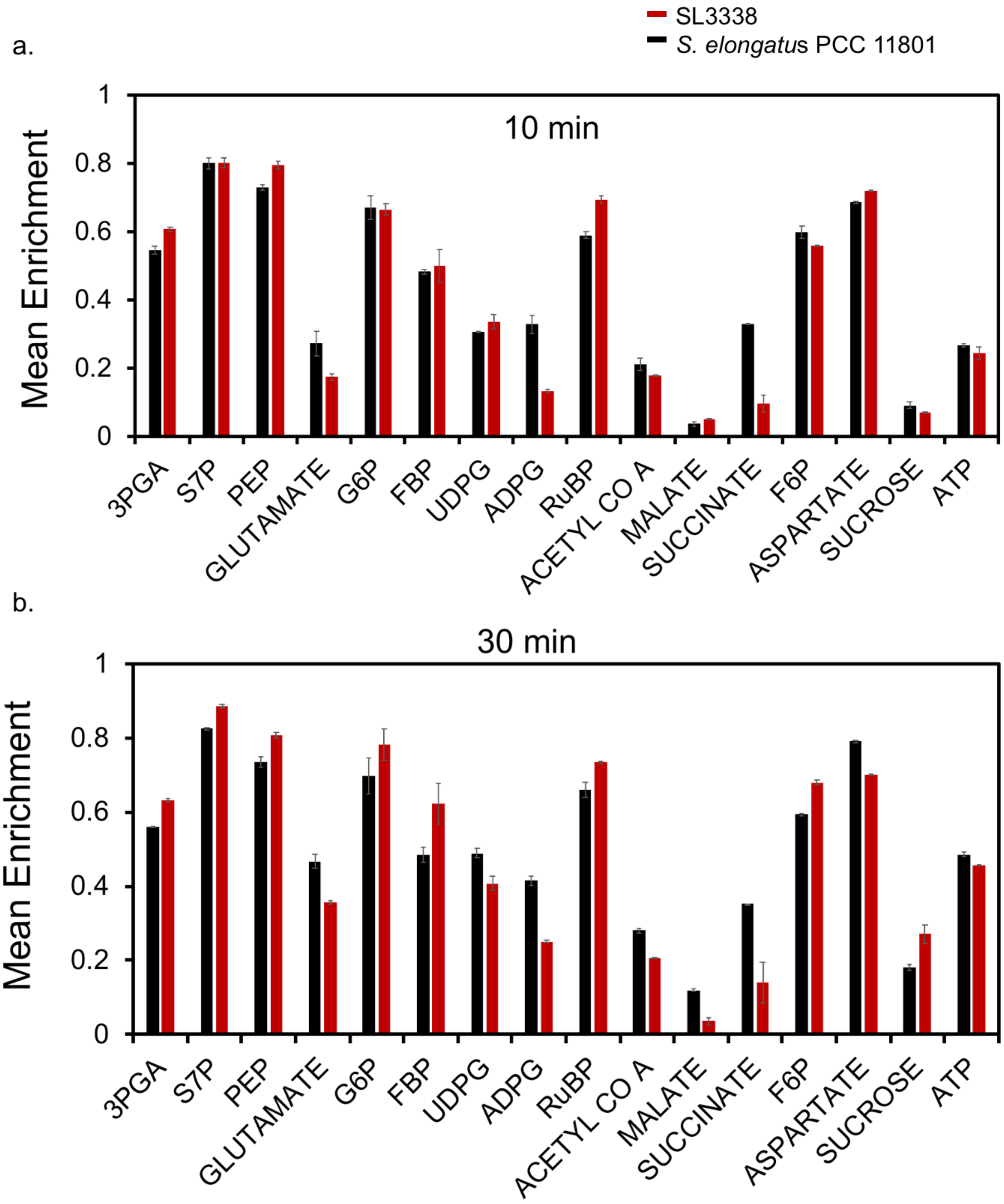

Figure S12: Comparison of mean enrichment of metabolites between the wild type and recombinant strains after (a) 10 min and (b) 30 mins. While the red bar indicate the recombinant, black is for wild type strain. Average of two biological replicates (each biological replicate having two technical replicates) was plotted. Error bars shown correspond to the standard error of mean (SEM) from the replicates (n=2). The abbreviations are provided in Figure S10. The SL3338 strain: *S. elongatus* PCC 11801-NSI::P_{cpcB}-*efe*-T_{rrnB}.

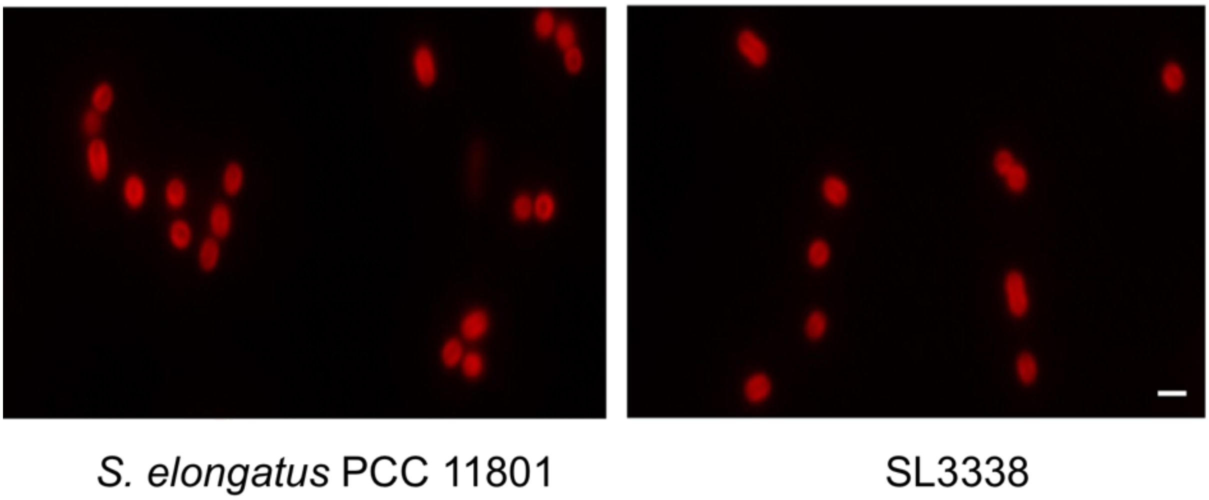

Figure S13: The microscopic image of the wild type and recombinant strains. No size or morphological difference was identified. SL3338: *S. elongatus* PCC 11801-NSI::P_{cpcB}-*efe*-T_{rrnB}

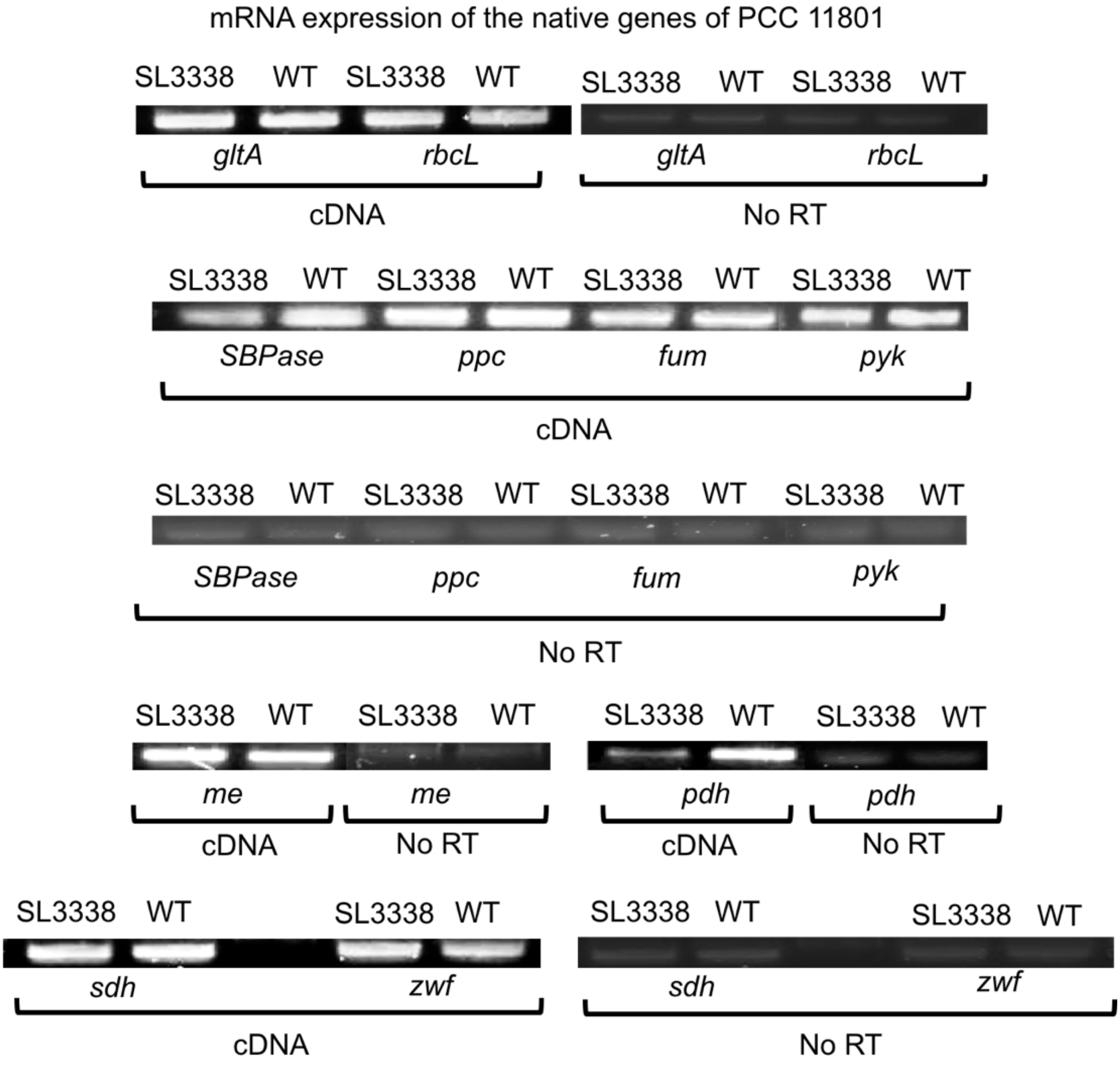

Figure S14: Qualitative RT-PCR was performed for both the wild type and recombinant strains showing the mRNA levels of the native genes present in the strains. The cDNA was prepared from the total RNA extracted from the strains and the primers specific for the gene was designed such that 200 bp was amplified in 30 PCR cycles. 16S rRNA was used as a loading control (Figure S2b). The abbreviations are: SL3338: *S. elongatus* PCC 11801-NSI::P_{cpcB}-*efe*-T_{rrnB} strain; WT: wild type *S. elongatus* PCC 11801; No RT: No Reverse Transcriptase enzyme was added to the PCR mixture and this acted as the ; *gltA*: Citrate synthase; *rbcL*: ribulose-bis-phosphate carboxylase/oxygenase large subunit; *SBPase*: sedoheptulose/fructose bisphosphatases; *ppc*: phosphoenolpyruvate carboxylase; *fum*: fumarase; *pyk*: pyruvate kinase; *me*: maleic enzyme; *sdh*: succinate dehydrogenase; *zwf*: Glucose-6-phosphate 1-dehydrogenase; *pdh*: pyruvate dehydrogenase.

Table S1: The list of primers employed in the study.

**Primer description**	**Primer sequence**
gRNA-NSI-fw	agatcagaagctggagtctctg
gRNA-NSI-rv	agacagcagagactccagcttctg
NS1a_Left	catttttttgtctagctttaatgcggtagttGGTACCcaacgcctattccaagggcg
NS1b_Right	gcccggattacagatcctctagagtcgacGGTACCctctctgaggggatgcagtgct
NS1b_left	ggctcagtcgaaagactgggccttctatcgggcaccgtaccgga
NS1a_cpc_Right	cggccacaagattcgctaaaaattgtgcaggcttctgccagtcgctt
cpcB_left	aagcgactggcagaagcctgcacaatttttagcgaatcttgtggccg
cpcB_right	caaaggtctgcaggttggtcattcaaccagtctcctgttctcgacttttat
efe_cpc_left	ataaaagtcgagaacaggagactggttgaatgaccaacctgcagacctttg
efe_right	gcgctactgccgccaggcactacttatcgtcgtcatccttgtaatcaatatcg
rrnB_left	caaggatgacgacgataagtagtgcctggcggcagtagcgc
rrnB_right	tccggtacggtgcccgatagaaggcccagtctttcgactgagcc
RT-efe-fw	attggcgctcacaccgatta
RT-efe-rv	tatcaccgggaaacacggtc
NSI-CF-fw	caacgcctattccaagggcg
NSI-CF-rv	ctctctgaggggatgcagtgct
RT-gltA_11801-fw	gatccctacgctgtagtcgc
RT-gltA_11801-rv	ctgcccgtggatctttgact
RT-rbcL_11801-fw	cgccaaatcgctgctcaaat
RT-rbcL_11801-rv	agccagcaacgcggatatag
RT-sbpase_11801-fw	gcgggctatgtcctctatgg
RT-sbpase_11801-rv	tcccggatgtactggcgata
RT-ppc_11801-fw	ggaaatccacaaggcccaga
RT-ppc_11801-rv	aggcagggcatatttcgagg
RT-fum_11801-fw	gcagtcacaaatggcagacc
RT-fum_11801-rv	tgccattaacgctgggacaa
RT-pyk_11801-fw	cttccatctgctcgatcgct
RT-pyk_11801-rv	atctgtccgtcaaagccctg
RT-me_11801-fw	gtcgagacgatgaagcggat
RT-me_11801-rv	cgtacctgcgacagggattt
RT-sdh_11801-fw	gatcgccttcgaccatgact
RT-sdh_11801-rv	gcactgcgatcgacgactaa

RT-zwf_11801-fw	ccggcgttttcacctcaaag
RT-zwf_11801-rv	gatggtcgataactggcgct
RT-pdh_11801-fw	gccttatccaacgggcagat
RT-pdh_11801-rv	tgaacactcccaacgactgg
RT-16s_11801-fw	agccacactgggactgagac
RT-16s_11801-fw	ttccctgagaaaaggggttt

Table S2: Detailed comparison of succinate and ethylene productivity reported in the literature for different cyanobacteria

SL. No.	Year	Organisms	Genetic Modifications	Growth/Production Condition	Reported Titers	OD	peak Ethylene productivity (µmole/g/h)	peak Succinate productivity (mg/g/h)	Ref
1	2013	*Synechocystis* sp. PCC 6803	*2Xefe*	5% CO_2, 20 mM $NaHCO_3$, 600 µE	250 µl/l/h/OD	-	44	---	(Ungerer et al., 2012)
2	2015	*Synechocystis* sp. PCC 6803	*1Xefe*, modified RBS	5% CO_2, 20 mM $NaHCO_3$, 50 µE	718 µl/l/h/OD	-	128.21	---	(Xiong et al., 2015)
3	2015	*Synechocystis* sp. PCC 6803	*	5% CO_2, 30 µE	9700 µl/l/h	20	86.61	---	(Zhu et al., 2015)
4	2015	*Synechocystis* sp. PCC 6803	Δ*ackA* and O.E.(*SigE*)	Dark anaerobic	120 mg/L in 5 days	20	---	0.2	(Osanai et al., 2015)
5	2016	*S. elongatus* PCC 7942	O.E.(*gabD*/*kgd*/*ppc*/*gltA*)	30µE, 50 mM $NaHCO_3$	80 mg/L/day	1.25	---	10.67	(Lan and Wei, 2016)
6	2017	*Synechocystis* sp. PCC 6803	#	Air, 50-100 µE	2463 µl/l/h/OD	-	439	---	(Mo et al., 2017)
7	2017	*Synechocystis* sp. PCC 6803	slr0168:: Ptrc-Rbs30-*efe*-Km^r	1% CO_2, 220 µE	443 µl/l/h	5	15.82	---	(Veetil et al., 2017)
8	2018	*Synechocystis* sp. PCC 6803	O.E.(*ppc*) and Δ*ackA*	Dark anaerobic	1.8 g/L in 72 h	25[a] g/L	---	1.0	(Hasunuma et al., 2018)
9	2019	*S. elongatus* PCC 7942	NSI:: Ptrc-*efe*-TrrnB	1% CO_2, 50 µE	140 µl/l/h/OD	-	25	---	(Carbonell et al., 2019)
10	2020	*Synechocystis* sp. PCC 6803	PnrsB-*efe*, O.E.(2X*ppc*, *ppsA*$_{7002}$)	50 mM $NaHCO_3$, 20 µE	20 µg/ml/OD /day	-	118.84	---	(Durall et al., 2020)
11		*S. elongatus* PCC 11801	NSI::$P_{cpcB300}$-*efe*-T_{rrnB}	LL-HC[b]	-	-	338.26	121.1	This study

a biomass used

b 20µE light with 1% CO_2 (chamber)in shake flask, or 1% CO_2 (bubbled) in MC 1000-OD or 50 mM $NaHCO_3$ in air-tight bottle

* slr0168::Sp^r-PcpcB-*efe*/sll1981::Km^r-PcpcB-*efe*/slr0370::Cm^r-PcpcB-*efe*/phaAB::Gm^r-pcpcB-*kgtP*

# slr0168::Sp^r-PcpcB-*efe*/sll1981::Km^r-PcpcB-*efe*/slr0370::Cm^r-PcpcB-*efe*/sll1423::Gm^r-pcpcB-*efe*

Table S3: The Quantum efficiency (Fv/Fm) of the wild type and recombinant strains measured using Phyto-PAM (Walz, Germany). SL3338: *S. elongatus* PCC 11801-NSI::P_{cpcB}-*efe*-T_{rrnB}. The data is from three biological replicates (each biological replicate having three technical replicates). Error bars shown correspond to SEM from the averaged biological replicates (n=3).

	WT-11801	SL3338	P-value
Fv/Fm	0.52±0.12	0.47±0.09	0.49
ETR max	25.9±1.7	21.1±3.5	0.288

Table S4: List of metabolites analyzed in the study

	Abbreviation	Compound Name	Formula	m/z Da	RT (min)	KEGG ID
**1**	GLY	Glycine	$C_2H_5NO_2$	74.03	1.1	C00037
**2**	GABA	Gamma-aminobutyric acid	$C_4H_9NO_2$	102.06	6.0	C00334
**3**	SUCC	Succinate	$C_4H_6O_4$	117.02	13.3	C00042
**4**	Pro-GLU	Proglutamic acid	$C_5H_7NO_3$	128.04	6.0	C01879
**5**	ASP	Aspartate	$C_4H_7NO_4$	132.03	6.0	C00049
**6**	GLN	Glutamine	$C_5H_{10}N_2O_3$	145.09	1.2	C00064
**7**	GLU	Glutamate	$C_5H_9NO_4$	146.05	5.7	C00025
**8**	PEP	Phosphoenolpyruvate	$C_3H_5O_6P$	166.98	15.0	C00074
**9**	3PGA	3-phosphoglycerate	$C_3H_7O_7P$	184.99	14.7	C00197
**10**	N-Acetyl GLU	N-acetylglutamate	$C_7H_{11}NO_5$	188.06	13.6	C00624
**11**	G6P	Glucose-6-phosphate	$C_6H_{13}O_9P$	259.02	10.6	C00668
**12**	F6P	Fructose-6-phosphate	$C_6H_{13}O_9P$	259.02	10.9	C00085
**13**	6PG	6-phosphogluconate	$C_6H_{13}O_{10}P$	274.98	14.7	C04442
**14**	S7P	Sedoheptulose-7-phosphate	$C_7H_{15}O_{10}P$	289.03	10.9	C05382
**15**	N-Ac-Glu-6-P	N-acetylglucosamine-6-phosphate	$C_8H_{16}NO_9P$	300.03	11.7	C00357
**16**	RuBP	Ribulose 1,5 bisphosphate	$C_5H_{12}O_{11}P_2$	308.98	15.2	C01182
**17**	Deoxy-TMP	deoxy-thymidine monophosphate	$C_{10}H_{15}N_2O_8P$	321.07	12.9	C00364
**18**	CMP	Cytidine monophosphate	$C_9H_{14}N_3O_8P$	322.05	11.6	C00055
**19**	UMP	Uridine monophosphate	$C_9H_{13}N_2O_9P$	323.03	11.9	C00105
**20**	FBP	Fructose-1,6-bisphosphate	$C_6H_{14}O_{12}P_2$	338.96	15.1	C05378
**21**	SUC	Sucrose	$C_{12}H_{22}O_{11}$	341.11	1.5	C00089
**22**	AMP	Adenosine monophosphate	$C_{10}H_{14}N_5O_7P$	346.06	13.7	C00020
**23**	IMP	Inosine monophosphate	$C_{10}H_{13}N_4O_8P$	347.05	12.1	C00130
**24**	SBP	Sedoheptulose-1,7-bisphosphate	$C_7H_{16}O_{13}P_2$	369.01	15.2	C00447
**25**	GMP	Guanosine monophosphate	$C_{10}H_{14}N_5O_8P$	362.02	12.4	C00144
**26**	XMP	Xanthosine monophosphate	$C_{10}H_{13}N_4O_9P$	363.04	14.8	C00655

**27**	CDP	Cytidine diphosphate	$C_9H_{15}N_3O_{11}P_2$	402.04	13.7	C00112
**28**	UDP	Uridine diphosphate	$C_9H_{14}N_2O_{12}P_2$	403.00	14.8	C00015
**29**	SUCROSE-6-P	Sucrose-6-phosphate	$C_{12}H_{23}O_{14}P$	421.08	10.5	C16688
**30**	ADP	Adenosine diphosphate	$C_{10}H_{15}N_5O_{10}P_2$	426.02	14.9	C00008
**31**	GDP	Guanosine diphosphate	$C_{10}H_{15}N_5O_{11}P_2$	442.02	14.7	C01228
**32**	ATP	Adenosine triphosphate	$C_{10}H_{16}N_5O_{13}P_3$	505.99	15.3	C00002
**33**	GTP	Guanosine triphosphate	$C_{10}H_{16}N_5O_{14}P_3$	521.99	15.2	C00044
**34**	dTDP-rhamnose	deoxy-TDP-rhamnose	$C_{16}H_{26}N_2O_{15}P_2$	547.08	14.1	C03319
**35**	UDPX	UDP-xylose	$C_{14}H_{22}N_2O_{16}P_2$	535.04	13.1	C00190
**36**	UDPG	UDP-glucose	$C_{15}H_{24}N_2O_{17}P_2$	565.05	13.2	C00029
**37**	UDPGluc	UDP-glucuronate	$C_{15}H_{22}N_2O_{18}P_2$	579.03	15.0	C00167
**38**	ADPG	ADP-glucose	$C_{16}H_{25}N_5O_{15}P_2$	588.08	14.1	C00498
**39**	GDP-fucose	GDP-fucose	$C_{16}H_{25}N_5O_{15}P_2$	588.08	13.6	C00325
**40**	GDP-mannose	GDP-mannose	$C_{16}H_{25}N_5O_{16}P_2$	604.07	13.1	C00096
**41**	UDP-GlcNAc	UDP-N-acetyl-glucosamine	$C_{17}H_{27}N_3O_{17}P_2$	606.08	13.1	C00043
**42**	GSSG	Oxidized glutathione	$C_{20}H_{32}N_6O_{12}S_2$	611.15	12.4	C00127
**43**	CMP-NAc-neuramininate	Cytidine monophosphate N-acetylneuraminic acid	$C_{20}H_{31}N_4O_{16}P$	613.15	12.5	C00128
**44**	ADP-ribose CP	ADP ribose 1,2 cyclic phosphate	$C_{15}H_{22}N_5O_{16}P_3$	620.02	15.0	C19851
**45**	NAD+	Nicotinamide adenine dinucleotide	$C_{21}H_{28}N_7O_{14}P_2$	663.11	10.3	C00003
**46**	UDP-NAc-muraminate	UDP-N-acetylmuraminate	$C_{20}H_{31}N_3O_{19}P_2$	678.10	15.1	C01050
**47**	NADP+	Nicotinamide adenine dinucleotide phosphate (NADP+)	$C_{21}H_{29}N_7O_{17}P_3$	743.08	14.9	C00003
**48**	FAD	Flavin adenine dinucleotide	$C_{27}H_{33}N_9O_{15}P_2$	784.15	15.3	C00016
**49**	ACETYL CoA	Acetyl Coenzyme A	$C_{23}H_{38}N_7O_{17}P_3S$	808.13	15.6	C00024
**50**	3-oxohexanoyl CoA	3-oxohexanoyl coenzyme A	$C_{27}H_{44}N_7O_{18}P_3S$	878.18	15.5	C05269
**51**	---	Glycogen	$C_{24}H_{42}O_{21}$	Not detected	---	C00182

Supplemental File:

1. Supplementary File S2: Table S5: The area ratios ($^{12}C/^{13}C$) for all the metabolites in the wild type and SL3338 strain of *S. elongatus* PCC 11801 under early phase (EP) (XLSX); Table S6: The area ratios ($^{12}C/^{13}C$) for all the metabolites in the wild type and SL3338 strain of *S. elongatus* PCC 11801 under late phase (EP) (XLSX).

2. Supplementary File S3: Mass isotopologoue distributions (MIDs) of the metabolites (under study) observed in wild type *S. elongatus* PCC 11801 and SL3338 (XLSX).

3. Supplementary File S4: Mean enrichment of the metabolites (under study) observed in wild type *S. elongatus* PCC 11801 and SL3338 (XLSX).

References:

Carbonell, V., Vuorio, E., Aro, E.M., Kallio, P., 2019. Enhanced stable production of ethylene in photosynthetic cyanobacterium *Synechococcus elongatus* PCC 7942. World J. Microbiol. Biotechnol. 35, 1–9. https://doi.org/10.1007/s11274-019-2652-7

Durall, C., Lindberg, P., Yu, J., Lindblad, P., 2020. Increased ethylene production by overexpressing phosphoenolpyruvate carboxylase in the cyanobacterium *Synechocystis* PCC 6803. Biotechnol. Biofuels 13, 16. https://doi.org/10.1186/s13068-020-1653-y

Hasunuma, T., Matsuda, M., Kato, Y., Vavricka, C.J., Kondo, A., 2018. Temperature enhanced succinate production concurrent with increased central metabolism turnover in the cyanobacterium *Synechocystis* sp. PCC 6803. Metab. Eng. 48, 109–120. https://doi.org/10.1016/j.ymben.2018.05.013

Lan, E.I., Wei, C.T., 2016. Metabolic engineering of cyanobacteria for the photosynthetic production of succinate. Metab. Eng. 38, 483–493. https://doi.org/10.1016/j.ymben.2016.10.014

Mo, H., Xie, X., Zhu, T., Lu, X., 2017. Effects of global transcription factor NtcA on photosynthetic production of ethylene in recombinant *Synechocystis* sp. PCC 6803. Biotechnol. Biofuels 10, 1–13. https://doi.org/10.1186/s13068-017-0832-y

Osanai, T., Shirai, T., Iijima, H., Nakaya, Y., Okamoto, M., Kondo, A., Hirai, M.Y., 2015. Genetic manipulation of a metabolic enzyme and a transcriptional regulator increasing succinate excretion from unicellular cyanobacterium. Front. Microbiol. 6, 1–10. https://doi.org/10.3389/fmicb.2015.01064

Ungerer, J., Tao, L., Davis, M., Ghirardi, M., Maness, P.C., Yu, J., 2012. Sustained photosynthetic conversion of CO2 to ethylene in recombinant cyanobacterium *Synechocystis* 6803. Energy Environ. Sci. 5, 8998–9006. https://doi.org/10.1039/c2ee22555g

Veetil, V.P., Angermayr, S.A., Hellingwerf, K.J., 2017. Ethylene production with engineered *Synechocystis* sp PCC 6803 strains. Microb. Cell Fact. 16, 1–11. https://doi.org/10.1186/s12934-017-0645-5

Xiong, W., Morgan, J.A., Ungerer, J., Wang, B., Maness, P.-C., Yu, J., 2015. The plasticity of cyanobacterial metabolism supports direct CO_2 conversion to ethylene. Nat. Plants 1, 15053. https://doi.org/10.1038/nplants.2015.53

Zhu, T., Xie, X., Li, Z., Tan, X., Lu, X., 2015. Enhancing photosynthetic production of ethylene in genetically engineered *Synechocystis* sp. PCC 6803. Green Chem. 17, 421–434. https://doi.org/10.1039/c4gc01730g
